# A blended genome and exome sequencing method captures genetic variation in an unbiased and cost-effective manner

**DOI:** 10.1038/s41588-026-02669-w

**Published:** 2026-07-08

**Authors:** Toni A. Boltz, Benjamin B. Chu, Matthew DeFelice, Calwing Liao, Julia M. Sealock, Robert Ye, Jacqueline I. Goldstein, Lerato Majara, Jack M. Fu, Susan K. Service, Lingyu Zhan, Sarah E. Medland, Sinéad B. Chapman, Simone Rubinacci, Jonna L. Grimsby, Tamrat Abebe, Melkam Alemayehu, Fred K. Ashaba, Elizabeth G. Atkinson, Tim B. Bigdeli, Amanda B. Bradway, Harrison Brand, Lori B. Chibnik, Samuel DeLuca, Ana M. Diaz-Zuluaga, Abebaw Fekadu, Michael Gatzen, Bizu Gelaye, Stella Gichuru, Marissa L. Gildea, Toni C. Hill, Hailiang Huang, Kalyn M. Hubbard, Wilfred E. Injera, Roxanne James, Moses Joloba, Christopher Kachulis, Phillip R. Kalmbach, Rogers Kamulegeya, Gabriel Kigen, Soyeon Kim, Nastassja Koen, Edith K. Kwobah, Joseph Kyebuzibwa, Seungmo Lee, Niall J. Lennon, Penelope A. Lind, Esteban A. Lopera-Maya, Johnstone Makale, Serghei Mangul, Justin McMahon, Pierre Mowlem, Henry Musinguzi, Rehema M. Mwema, Noeline Nakasujja, Carter P. Newman, Lethukuthula L. Nkambule, Conor R. O’Neil, Ana Maria Olivares, Catherine M. Olsen, Linnet Ongeri, Sophie J. Parsa, Adele Pretorius, Shengying Qin, Raj Ramesar, Faye L. Reagan, Chiara Sabatti, Jacquelyn A. Schneider, Welelta Shiferaw, Christine Stevens, Anne Stevenson, Erik Stricker, Rocky E. Stroud, Jessie Tang, Megan Townsend, David Whiteman, Mary T. Yohannes, Mingrui Yu, Kai Yuan, NeuroGAP-Psychosis Study, Anne Stevenson, Rocky E. Stroud, Dickens Akena, Lukoye Atwoli, Symon M. Kariuki, Karestan C. Koenen, Charles R. J. C. Newton, Dan J. Stein, Solomon Teferra, Zukiswa Zingela, Dickens Akena, Lukoye Atwoli, Symon M. Kariuki, Karestan C. Koenen, Charles R. J. C. Newton, Dan J. Stein, Solomon Teferra, Zukiswa Zingela, Carlos N. Pato, Michele T. Pato, Carlos Lopez-Jaramillo, Nelson B. Freimer, Roel A. Ophoff, Loes M. Olde Loohuis, Michael E. Talkowski, Benjamin M. Neale, Daniel P. Howrigan, Alicia R. Martin

**Affiliations:** 1 Stanley Center for Psychiatric Research, Broad Institute of MIT and Harvard, Cambridge, MA USA; 2 Analytic and Translational Genetics Unit, Department of Medicine, Massachusetts General Hospital, Boston, MA USA; 3 Department of Biomedical Data Sciences, Stanford University, Stanford, CA USA; 4 Broad Clinical Labs, Broad Institute of MIT and Harvard, Cambridge, MA USA; 5 Program in Medical and Population Genetics, Broad Institute of MIT and Harvard, Cambridge, MA USA; 6 Department of Psychiatry and Mental Health and South African Medical Council Research Unit on Risk and Resilience in Mental Disorders, Neuroscience Institute, University of Cape Town and Groote Schuur Hospital, Cape Town, South Africa; 7 Department of Neurology, Massachusetts General Hospital and Harvard Medical School, Boston, MA USA; 8 Center for Neurobehavioral Genetics, Semel Institute for Neuroscience and Human Behavior, University of California Los Angeles, Los Angeles, CA USA; 9 The Collaboratory, Institute for Quantitative and Computational Biosciences, University of California Los Angeles, Los Angeles, CA USA; 10 QIMR Berghofer Medical Research Institute, Brisbane, Queensland Australia; 11 BD2: Breakthrough Discoveries for thriving with Bipolar Disorder, Santa Monica, CA USA; 12 Division of Genetics, Department of Medicine, Brigham and Women’s Hospital and Harvard Medical School, Boston, MA USA; 13 Center for Data Sciences, Brigham and Women’s Hospital and Harvard Medical School, Boston, MA USA; 14 Department of Microbiology, Immunology, and Parasitology, School of Medicine, College of Health Sciences, Addis Ababa University, Addis Ababa, Ethiopia; 15 Department of Psychiatry, School of Medicine, College of Health Sciences, Addis Ababa University, Addis Ababa, Ethiopia; 16 Department of Immunology and Molecular Biology, College of Health Sciences, Makerere University, Kampala, Uganda; 17 Department of Molecular and Human Genetics, Baylor College of Medicine, Houston, TX USA; 18 Dan Duncan Neurological Research Institute, Texas Children’s Hospital, Houston, TX USA; 19 Institute for Genomics in Health, The State University of New York, Brooklyn, NY USA; 20 Center for Genomic Medicine, Massachusetts General Hospital, Boston, MA USA; 21 Department of Neurology, Massachusetts General Hospital, Boston, MA USA; 22 Department of Epidemiology, Harvard T. H. Chan School of Public Health, Boston, MA USA; 23 Centre for Innovative Drug Development and Therapeutic Trials for Africa, Addis Ababa University, Addis Ababa, Ethiopia; 24 Epidemiology Branch, Division of Population Health Research, Division of Intramural Research, Eunice Kennedy Shriver National Institute of Child Health and Human Development, Bethesda, MD USA; 25 Department of Mental Health, Moi Teaching and Referral Hospital, Eldoret, Kenya; 26 Department of Medical Laboratory Sciences, School of Health Sciences, Alupe University, Busia, Kenya; 27 Department of Psychiatry and Mental Health, University of Cape Town, Cape Town, South Africa; 28 School of Biomedical Sciences, School of Medicine, College of Health Sciences, Makerere University, Kampala, Uganda; 29 Department of Pharmacology and Toxicology, Moi University School of Medicine, Eldoret, Kenya; 30 Department of Medicine, Kyung Hee University College of Medicine, Seoul, Republic of Korea; 31 SA MRC Unit on Risk and Resilience in Mental Disorders, University of Cape Town and Neuroscience Institute, Cape Town, South Africa; 32 Department of Psychiatry, School of Medicine, College of Health Sciences, Makerere University, Kampala, Uganda; 33 Department of Computer Science, University of California Los Angeles, Los Angeles, CA USA; 34 Department of Human Genetics, David Geffen School of Medicine, University of California Los Angeles, Los Angeles, CA USA; 35 Epidemiology and Demography Department, KEMRI-Wellcome Trust Research Programme-Coast, Kilifi, Kenya; 36 Małopolska Centre of Biotechnology, Jagiellonian University, Kraków, Poland; 37 Department of Biological and Morphofunctional Sciences, College of Medicine and Biological Sciences, Stefan cel Mare University of Suceava, Suceava, Romania; 38 Department of Computers, Informatics, and Microelectronics, Technical University of Moldova, Chisinau, Moldova; 39 Department of Clinical Pharmacy, Alfred E. Mann School of Pharmacy, University of Southern California, Los Angeles, CA USA; 40 Ampath Laboratories, Moi University School of Medicine, Eldoret, Kenya; 41 Neurosciences Unit, Clinical Department, KEMRI-Wellcome Trust Research Programme-Coast, Kilifi, Kenya; 42 Centre for Clinical Research, Kenya Medical Research Institute, Nairobi, Kenya; 43 Bio-X Institutes, Key Laboratory for the Genetics of Developmental and Neuropsychiatric Disorders, Shanghai Jiao Tong University, Shanghai, China; 44 MRC Genomic and Precision Medicine Research Unit, Division of Human Genetics, Institute of Infectious Diseases and Molecular Medicine, Faculty of Health Sciences, University of Cape Town, Cape Town, South Africa; 45 Department of Psychiatry, Faculty of Medicine and Health Sciences, Stellenbosch University, Cape Town, South Africa; 46 Department of Mental Health and Behavioural Sciences, School of Medicine, Moi University College of Health Sciences, Eldoret, Kenya; 47 Brain and Mind Institute, The Aga Khan University, Nairobi, Kenya; 48 Department of Medicine, Medical College East Africa, The Aga Khan University, Nairobi, Kenya; 49 Department of Psychiatry, University of Oxford, Oxford, UK; 50 Department of Social and Behavioral Sciences, Harvard T. H. Chan School of Public Health, Boston, MA USA; 51 Psychiatric and Neurodevelopmental Genetics Unit, Department of Psychiatry, Massachusetts General Hospital, Boston, MA USA; 52 Executive Dean’s Office, Faculty of Health Sciences, Nelson Mandela University, Gqebera, South Africa; 53 Rutgers University, New Brunswick, NJ USA; 54 Department of Psychiatry, University of Antioquia, University of Antioquia, Medellín, Antioquia, Colombia; 55 Department of Computational Medicine, David Geffen School of Medicine, University of California Los Angeles, Los Angeles, CA USA

**Keywords:** DNA sequencing, Population genetics

## Abstract

Here we developed and deployed the blended genome exome (BGE) method, a DNA library approach that generates low-pass whole-genome (1–4× mean depth) and deep whole-exome (30–40× mean depth) data in a single sequencing run. BGE is cost-effective, empowers most genomic discoveries possible with deep whole-genome sequencing and captures global common single-nucleotide polymorphism diversity. We applied BGE to sequence >53,000 samples from the PUMAS Project (Populations Underrepresented in Mental Illness Associations Studies), including African, African American and Latin American populations. Imputed genotypes showed high concordance with Illumina Global Screening Array calls (*R*^2^ ≥ 95% for minor allele frequency ≥1%; ≥90% for minor allele frequency <1%), with consistent performance across local ancestries in admixed cohorts. For protein-coding copy number variants, deletions and duplications spanning at least three exons had a positive predicted value of ~90% relative to deep whole-genome data. At ~28% of the cost of deep whole-genome sequencing, BGE provides a scalable, reliable platform to expand genomic discovery and equitable access to sequencing in underrepresented populations.

## Abstract (web summary)

Blended genome exome (BGE) is a sequencing method that captures genetic variation in an unbiased and cost-effective manner. Applying BGE to sequence samples from underrepresented populations leads to improved variant discovery.

## Main

Genome-wide association studies (GWAS) have grown exponentially over the past 15 years, rapidly increasing in statistical power to enable the identification of hundreds of thousands of associations between genetic variants and human traits^1^. While these discoveries have been facilitated in part by precipitous drops in sequencing costs, microarrays have been the primary technology used for GWAS to date because of their lower costs. However, by design, they have biased ascertainment of genetic variants; sites that are included on many GWAS arrays, such as the widely used Illumina Global Screening Array (GSA) or Global Diversity Array, are most common in European ancestry populations. Previous work has shown that low-coverage sequencing is a cost-effective alternative that can more accurately capture genetic variants across the allele frequency spectrum for variants present in imputation reference panels^2,3^. Low-coverage sequencing is especially useful in populations underrepresented in genomics, even compared with GWAS arrays that have been designed to reflect variation within those populations, such as the H3Africa GWAS Array^3^. Another study from Li et al.^4^ demonstrated that low-pass whole-genome sequencing at 0.5–1× coverage outperformed the Illumina GSA in imputation accuracy, resulting in greater power for GWAS and more reliable polygenic risk score estimates, particularly in populations of African ancestry where array-based ascertainment bias is most pronounced. Both genetic data generation strategies are useful for evaluating the architectures of complex traits and associating common variants with them.

High-coverage genome sequencing, while more expensive, captures a more complete spectrum of genetic variation. Balancing its higher cost with the need for large sample sizes to achieve robust disease and trait associations, researchers often sequence only coding regions using an exome capture, typically to high coverage (~60×), and supplement with GWAS arrays. This strategy is useful for prioritizing genes, as rare coding variants currently comprise most of the known variants that have interpretable functions and are therapeutically actionable^5–7^. Combining exome sequencing with GWAS arrays enables researchers to glean analytical insights from both common variant GWAS and rare variant tests, including those that assess gene burden and those that evaluate associations to individual variants. Another recent study has shown the utility of conducting high-coverage exome sequencing in parallel with low-coverage whole-genome sequencing (WGS) to gain additional, non-imputable disease associated variants available to be evaluated^8^. To increase the cost efficiency and scalability of a combined strategy, we developed a new blended genome exome (BGE) approach. BGE sequences the whole genome to at least 1–4× depth and the exome at ≥30× depth, reducing costs. Another benefit of BGE compared with disjoint exome plus GWAS array or low-coverage sequencing on the same samples is the unified protocol that streamlines comparisons between imputed versus high-quality and high-coverage coding variants across the allele frequency spectrum and thus enables improved quality control (QC). However, it requires the development of new computational approaches and pipelines as well as haplotype reference panels that can support genotype calling and refinement.

Here, we demonstrate the utility of BGE across diverse populations that have been underserved with traditional GWAS arrays by applying BGE at scale by sequencing samples from the PUMAS Project. PUMAS is an umbrella project consisting of multiple substudies, including: the Genomic Psychiatry Cohort (GPC), which primarily includes admixed African American and Hispanic/Latino populations from the USA; the NeuroGAP-Psychosis (Neuropsychiatric Genetics in African Populations-Psychosis) study, consisting of participants from Ethiopia, Kenya, South Africa and Uganda; and the Paisa Study, consisting of participants from a recently admixed population in Colombia. We report high genetic data quality at a relatively low cost when applying the BGE method to sequence 53,448 samples from the above populations. To facilitate benchmarks against gold-standard data, we also applied BGE sequencing to 400 individuals enrolled with a family study design from the Simons Simplex Collection (SSC) provided from the Simons Foundation Autism Research Initiative^9^, which had previously been sequenced to high coverage to evaluate recall and positive predictive value (PPV) for exonic copy number variants (CNVs) and structural variants. We also evaluate imputation concordance of BGE using an orthogonal data generation method, standard GWAS arrays, in a subset of the same PUMAS participants; we stratified by local ancestry where applicable. To support broader adoption, we provide a resource of open-source scripts and analytical pipelines for conducting these analyses with BGE (see ‘Code availability’ section), empowering other sequencing centers and analysts to apply this new strategy to cost-effectively improve variant discovery, especially in underrepresented populations.

## Results

### The BGE protocol balances cost with genotype quality

**Fig. 1:**
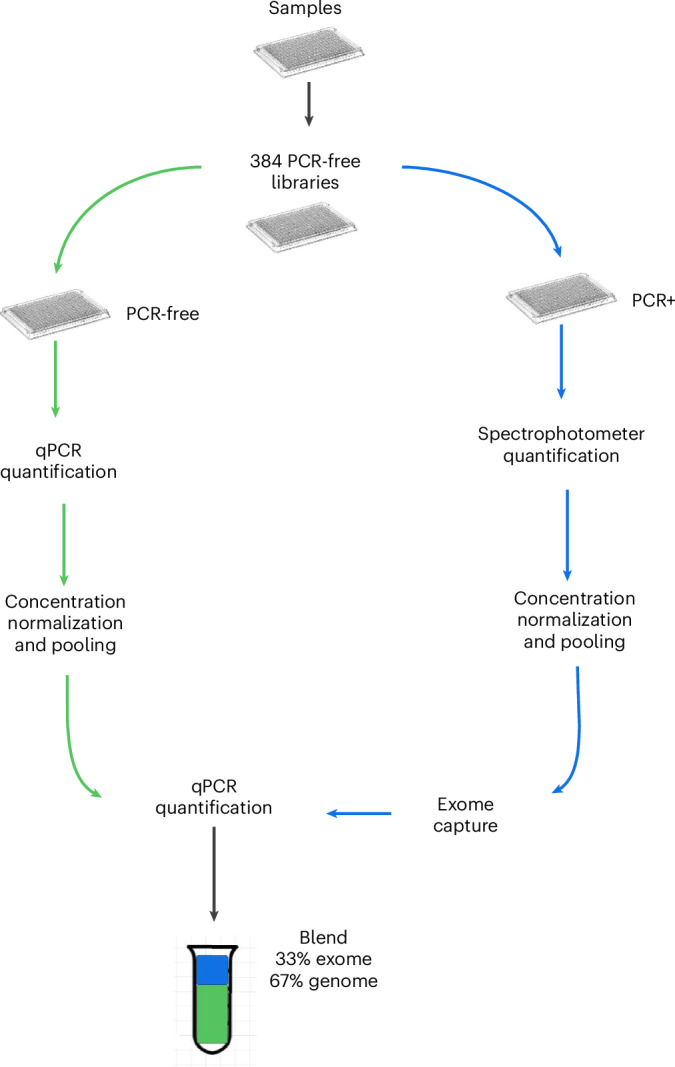
BGE lab protocol. In the BGE lab process, PCR-free libraries are constructed from genomic DNA, quantified by quantitative PCR (qPCR), normalized and pooled. An aliquot of the individual PCR-free libraries is PCR amplified, and PCR+ libraries are quantified by spectrophotometer, normalized and pooled. The PCR+ library pool undergoes exome capture, is enriched and quantified by qPCR. The PCR-free pool and the PCR+ pool are blended (33% exome, 67% genome) and sequenced on NovaSeqS4 (equivalent of 64 samples per lane).

To develop the BGE protocol (Fig. 1), we performed six iterative rounds of experimentation before implementing BGE at a large scale to determine the best blending ratio of whole-exome sequencing (WES) to WGS. The amount of sequencing required per sample sought to achieve the following objective: cost-efficiently maintaining >99% imputation *R*^2^ nonreference concordance for imputed variants with minor allele frequency (MAF) >5% compared with existing 30× WGS while providing enough exome coverage for 90% of exome bases to reach 10× depth (Supplementary Methods). The first round tested 96 samples per lane of NovaSeqS4 (Illumina) and nanomolar blending of 67% WES:33% WGS. This ratio generated 29× WES and 1.5× WGS coverage on average per sample and resulted in >99% *R*^2^ concordance of BGE-imputed variants in the exome, but <99% for whole-genome variants in samples with lower coverage. In the subsequent experiments (rounds 2–6), we titrated the WES:WGS blending ratio and sequencing coverage to determine the optimal protocol for achieving >99% *R*^2^ concordance for calling both exome and genome variants while minimizing cost. Table 1 presents the blending ratios, amount sequenced and resulting WES and WGS coverage across rounds.

**Table 1:** Iterative rounds of BGE development

Round	1	2	3	4	5	6 – Scaling
Blending ratio	67% WES + 33% WGS	67% WES + 33% WGS	60% WES + 40% WGS	40% WES + 60% WGS	33% WES + 67% WGS	33% WES + 67% WGS
Amount of sequencing (average Gb per sample)	8.2 Gb per sample	18.5 Gb per sample	18.7 Gb per sample	18.5 Gb per sample	12 Gb per sample	12 Gb per sample
Coverage (mean)	29× WES 1.5× WGS	78× WES 2.55× WGS	78× WES 2.5× WGS	64.8× WES 3.7× WGS	37.7× WES 2.37× WGS	37× WES 2.5× WGS
Exome bases ≥10× (%)	96.33	98.34	98.27	98.18	96.44	96.37
Exome bases ≥20× (%)	75.09	97.84	97.79	97.19	87.04	84.08
Genotype concordance *R*^2^ median (s.d.)	0.988 (1.56 × 10^−3^)	0.991 (1.28 × 10^−3^)	0.990 (2.90 × 10^−3^)	0.995 (3.55 × 10^−2^)	0.993 (1.15 × 10^−3^)	0.993 (1.05 × 10^−3^)

Per-sample genotype concordance between deep whole-genome variants and filtered Haplotype Reference Consortium (HRC)-imputed variants from the low-pass genome. Rounds 1 to 5 were limited to 68 samples from the Broad Institute Study of Inflammatory Bowel Disease Genetics. Round 6 tested concordance on 22 samples drawn from the PUMAS NeuroGAP-Psychosis sample set described in the Supplementary Methods.

To achieve optimal blending and sequencing depth, we found that 33% WES and 67% WGS for 64 samples per lane provided adequate coverage of each (30–40× WES and 1–4× WGS per sample) for calling variants, and >99% *R*^2^ concordance to 30× WGS common variants (MAF > 5%) for both the exome and imputed genome. All BGE blending ratios tested provided better *R*^2^ concordance to 30× WGS data than the GSA (Supplementary Fig. 1). We recommend using the following target metrics for BGE sequencing: (1) >9.5 Gb total bases per sample passing Illumina’s pass filter, (2) >90% of exome bases at 10× coverage, and (3) between 10% and 65% of reads on/near exome capture regions (within 250–500 bp of the probe). These deliverables provide adequate exome coverage for rare variant detection and genome coverage for common variant imputation.

### BGE QC at scale on diverse participants

**Fig. 2:**
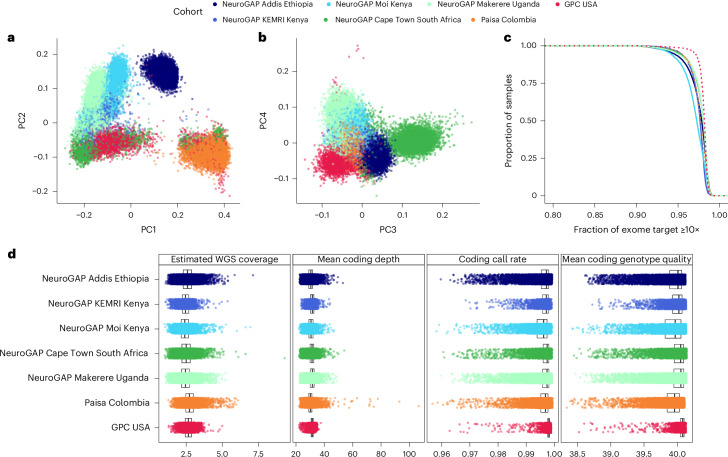
Expected ancestral diversity, coverage and quality from BGE data at scale. **a**,**b**, Principal component 1 (PC1) versus PC2 (**a**) and PC3 versus PC4 (**b**). **c**, Fraction of exome target covered with at least 10× depth stratified by cohort and collection method. Solid lines indicate saliva collection (NeuroGAP), and dashed lines indicate blood collection (GPC and Paisa). **d**, Estimated mean WGS coverage, mean coding depth, mean coding call rate and mean coding genotype quality. The boxplots display the median as the central horizontal line, with the lower and upper edges of the box representing the first (Q1) and third (Q3) quartiles, respectively.

With our recommended BGE protocol in place, we sequenced 53,446 individuals (Table 2) from an ancestrally diverse set of participants collected in the PUMAS Project (Fig. 2a,b, Supplementary Fig. 2 and Methods). More details on the ancestry composition of these cohorts are presented in Supplementary Table 2. Exome coverage differed based on DNA collection method, with blood samples from GPC and Paisa slightly outperforming saliva samples from the NeuroGAP-Psychosis (percent failure rates: GPC 0.04%, Paisa 0.13%, NeuroGAP-Psychosis 0.48%; Fig. 2c). However, across all cohorts, the percentage of samples failing to meet the exome coverage threshold was consistently less than 1%, with only six samples removed for having less than 1× average WGS coverage (Fig. 2d). Overall, the exome portion of the BGE demonstrated robust performance, with mean call rates >0.99, mean read depth >30 and mean genotype quality >39 across all cohorts (Fig. 2d). We defined high-quality samples using the exome portion of the BGE and continental ancestry strata-based filtering, which retained 88% of the samples (Table 2, Supplementary Information and Methods).

**Table 2:** Cohorts included throughout analyses

Cohort name and location	Number of total pre-QC	Number of total post-QC
NeuroGAP Addis Ethiopia	11,715	11,027
NeuroGAP KEMRI Kenya	3,078	2,889
NeuroGAP Moi Kenya	5,040	4,716
NeuroGAP Cape Town South Africa	8,747	5,779
NeuroGAP Makerere Uganda	11,306	10,727
Paisa Colombia	9,007	8,200
GPC USA	4,553	3,926
Total	53,446	47,264

QC includes filtering on WES and WGS coverage, genetic ancestry, outlier filtering on sample quality metrics, and checking for discrepancies between genetic sex and reported gender.

### Accurate CNV and structural variant discovery

We examined the potential of BGE for detecting CNVs, focusing initially on the higher coverage exome sequences. CNV discovery from WES data has traditionally been challenging owing to the highly variable exome capture efficiency between different capture kits and sequencing centers, as well as the complexities introduced by using read-depth information to infer copy state from short-read sequencing data. These factors have most often resulted in restricting exome analyses of SNVs and indels, despite the considerable value of capturing and predicting the functional impact of CNVs that can alter gene dosage and/or disrupt normal gene function. Recently, members of our group have published the GATK-gCNV algorithm^10^, which is a read-depth based method built on a hierarchical hidden Markov model that reliably detects rare CNVs overlapping coding exons. GATK-gCNV adjusts for known WES read-depth confounders such as sequence composition and mappability of exon bins, while also adjusting for unspecified technical batch confounders. This algorithm is able to achieve high recall (>90%) from standard WES by comparison with exonic CNVs captured by deep WGS, while also dramatically reducing false-positive discoveries to a level appropriate for stringent variant association testing.

**Fig. 3:**
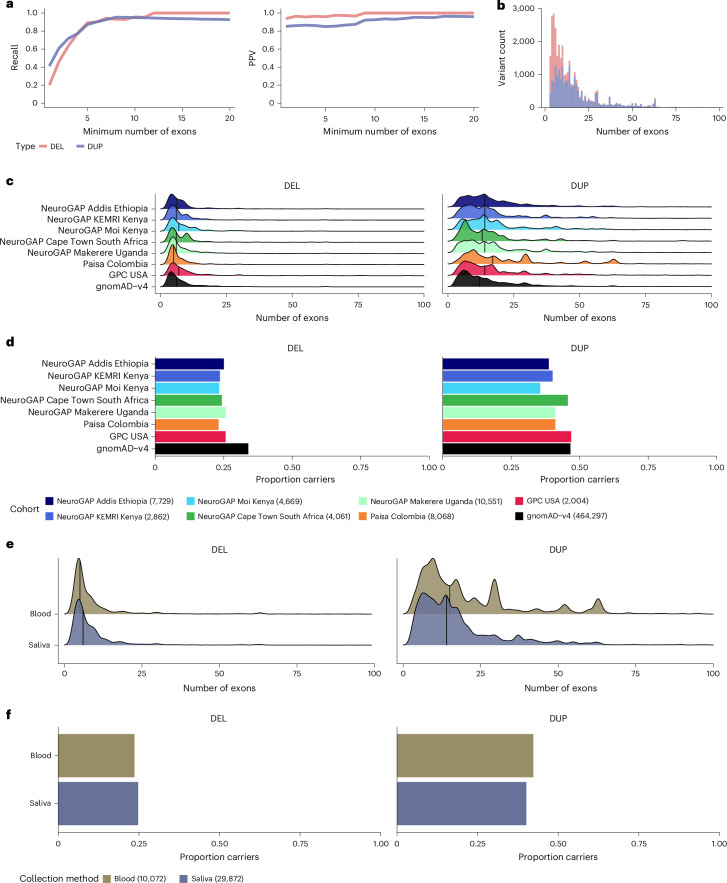
Protein-coding CNVs have expected qualities in BGE compared with WGS. **a**, Recall and PPV of CNVs called from the BGE relative to matched WGS samples (*N* = 400). **b**, Distribution of deletions and duplications across all cohorts. **c**, Distribution of CNV sizes across cohorts by number of exons. **d**, Proportion of unique deletion (DEL) and duplication (DUP) carriers across cohorts. **e**, Comparison of CNV size across cohorts by number of exons for saliva and blood. **f**, Comparison of unique deletion and duplication carriers between blood and saliva.

To evaluate GATK-gCNV performance in the exome portion of the BGE data, we applied it to 400 familial samples from the SSC, an autism cohort provided by the Simons Foundation Autism Research Initiative^9^. All samples have orthogonal validation data available, including high-coverage whole-genome sequencing, exome sequencing and matched microarray data. These samples represented 400 individuals from 100 quartet families, each with two children and both parents, providing a robust design for benchmarking and evaluating de novo and transmitted events. We generated BGE data for these samples and applied GATK-gCNV to the exome regions for CNV detection following the published exome CNV parameters^10^. Using gold-standard CNV calls from high-coverage WGS data of these samples, we achieved 87% recall of CNVs spanning five or more exons (Fig. 3a). Recall was slightly lower with BGE compared with standard WES because of lower exome coverage (<60× in BGE versus >60× for previously benchmarked WES). Focusing on only the samples with higher-coverage BGE (>60×) achieved 100% recall at five exons compared against our benchmark genomes. GATK-gCNV maintained a ~90% PPV at a resolution of three or more exons. Taking advantage of the complete family structure of the data, we also examined the de novo CNVs that were detected and found that 100% of the confirmed de novo CNVs (11 de novos) reported in the gold-standard WGS CNV study were also detected in BGE data, with no additional false-positive de novo CNVs predicted by our application of GATK-gCNV to BGE data.

We applied GATK-gCNV to all BGE samples that passed SNV QC. We found that there were more deletions than duplications, and duplications were larger than deletions on average (Fig. 3b), consistent with previous observations from WGS and WES studies^11^. Across cohorts, we found similar distributions of CNV size as measured by number of exons covered for both deletions and duplications (Fig. 3c). Finally, we compared whether CNV discovery with GATK-gCNV differed between blood and saliva, and found no meaningful differences for deletions and duplications in terms of unique carriers or CNV size (Fig. 3e,f). Similarly, when we restricted to both control samples and constrained genes across two different constraint metrics (LOEUF^12^ and GISMO-mis^13^), we did not find significant evidence for more CNVs in blood than saliva samples (odds ratio 1.2001, *P* = 0.1243 for LOEUF*;* odds ratio 1.2625, *P* = 0.0446 for GISMO-mis) (Supplementary Fig. 3).

We additionally evaluated the feasibility of calling structural variants beyond coding regions alone. Specifically, we used two recently developed tools, VISTA (variant identification and structural variant analysis)^14^, which is an ensemble method combining structural variant callers optimized for a range of structural variant lengths and sequencing depths, and INSurVeyor^15^, a sensitive and precise insertion caller. We applied these tools to samples from the SSC cohort where both WGS and BGE data were available (*N* = 395). We treated high-coverage WGS data as the gold standard and compared the deletion and insertion events identified from the two datasets. For insertions above 50 bp, the overall calling rate (*μ*_INS_BGE_ = 552.8 and *μ*_INS_WGS_ = 4,551.4) was lower than deletions (*μ*_DEL_BGE_ = 1,588.1 and *μ*_INS_WGS_ = 10,590.9). Nonetheless, we observed a high mean PPV score of 79.31% and a low mean recall score of 23.04% (Supplementary Fig. 4a,c). Similarly, we observed a high PPV (99.14% on average) but low recall (34.78% on average) for deletions (Supplementary Fig. 4b,d). The range of deletion event sizes identified by both platforms was also comparable, spanning 50–223,224,830 bp in the BGE data and 50–223,224,792 bp in the WGS data (Supplementary Fig. 5a,b).

### High concordance between imputed BGE and GWAS array data

We imputed BGE data directly from CRAM files with GLIMPSE2^16,17^ using a harmonized high-coverage WGS reference panel composed of the Human Genome Diversity Project and 1000 Genomes Project (HGDP+1kGP)^18^. In total, we imputed over 67 million bi-allelic single-nucleotide polymorphisms (SNPs) across the allele frequency spectrum. We filtered to high-quality variants, which we define as INFO score ≥0.8, resulting in over 30 million SNPs in downstream concordance analyses. Most of the SNPs removed owing to INFO-score filtering had MAF < 0.01 (Supplementary Table 3).

**Fig. 4:**
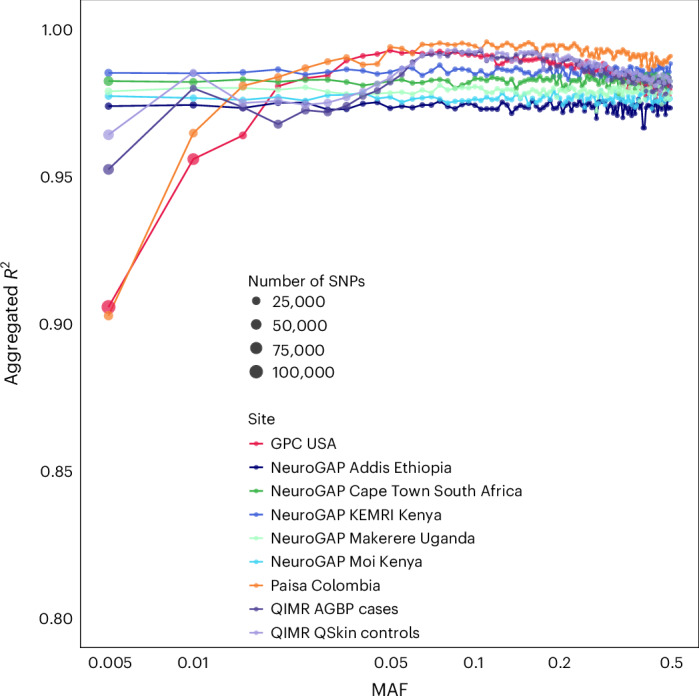
Imputation of BGE data is highly concordant with GWAS array data across MAF bins. The sizes of points correspond to numbers of SNPs in each MAF bin. Variants are filtered to those passing an INFO score ≥0.8. SNP MAFs are defined within cohorts using the GSA array for the Paisa, GPC and QIMR cohorts. Owing to limited GSA samples in the NeuroGAP-Psychosis cohorts, MAFs are defined using full cohort imputed data per site^18^.

Subsets of participants with BGE data from each cohort were also genotyped on the Illumina GSA. We evaluated imputation accuracy by comparing BGE imputed genotypes to unimputed GSA array-based genotypes that passed QC (Methods), providing an orthogonal comparison of genotypes (Supplementary Table 4). In this analysis, we evaluated two additional mostly European ancestry cohorts with BGE and GSA data available that are comparable with previous studies^16^. These two cohorts are from the Queensland Institute of Medical Research (QIMR), including the Australian Genetics of Bipolar Disorder Study^19^ (AGBP, *n* = 3,664 here) and the QSkin Sun and Health Study^20^ (*n* = 3,545 here). We computed Pearson’s aggregated *R*^2^ within each cohort, grouping SNPs by MAF bin. We found that all cohorts exceed 90% *R*^2^ across all MAF bins analyzed (Fig. 4), suggesting high accuracy of the imputed genotype dosages derived from BGE sequencing. We observed a slight downward trend in aggregated *R*^2^ at MAF > 0.05; however, accuracy remained high.

We also computed nonreference concordance values per cohort (Supplementary Fig. 6a) and found that, for common variants (MAF ≥ 1%), BGE data from all cohorts were accurately imputed with at least 0.92 nonreference concordance. For the lowest MAF bin (0–0.005), we find that all cohorts have a nonreference concordance of at least 0.88, suggesting that, although there is a slight drop in accuracy, most intermediate to common frequency variants are well imputed, in agreement with the aggregated *R*^2^. Across nearly all cohorts, exonic variants achieve higher concordance at lower allele frequencies than non-exonic sites. For instance, at a MAF of 0.01, exonic concordance in the NeuroGAP-Psychosis sites exceeds 0.95, whereas non-exonic concordance in the same cohorts only reaches 0.95 at a MAF of approximately 0.05 (Supplementary Fig. 6b,c). The one exception is at the very rarest bin (MAF of 0.005) in GPC, where non-exonic calls (0.90) slightly outperform exonic calls (0.875), probably reflecting stochasticity in the limited number of rare coding sites. Even for these variants, exonic accuracy exceeds non-exonic by the next frequency bin (MAF of 0.01). Paisa shows near-perfect concordance (>0.99) for exonic variants at a MAF as low as 0.01, whereas its non-exonic performance only reaches a similar plateau at MAF > 0.05. In summary, deeper exome coverage delivers superior call accuracy for rare coding SNPs, whereas low-coverage genome sequencing yields slightly lower concordance for ultrarare noncoding variants until they become more common (MAF of ~0.05).

In addition to INFO score, filtering on posterior genotype probabilities can improve these concordance metrics (Supplementary Fig. 7a,b) and attenuate the slight downward trend in aggregated *R*^2^ with a small fraction of dropped genotypes (Supplementary Fig. 7c). Overall, this analysis shows that imputed genotypes generated from low-pass WGS on the BGE platform are highly concordant with standard array-based genotypes.

### BGE imputation quality across ancestries

We further evaluated BGE imputation accuracy by dividing genotypes into different ancestry tracts based on their diploid local ancestry, following previous work^21^. We focus on the Paisa and GPC cohort here, as these populations have recently undergone global continental admixture. Using quality-controlled GWAS array genotype data, we inferred three-way local ancestry for Paisa and two-way local ancestry for GPC using RFMix2^22^. As reference panels for local ancestry inference, we used all individuals within the ancestry groups and labels provided by the HGDP+1kGP resource, as follows: AFR (African), AMR (Admixed American) and EUR (European populations) (AFR/AMR/EUR in three-way inference and AFR/EUR in two-way inference). We selected subsets of the HGDP+1kGP^18^ as reference panels for distinct source populations of admixture within Paisa and/or GPC cohorts (Methods).

**Fig. 5:**
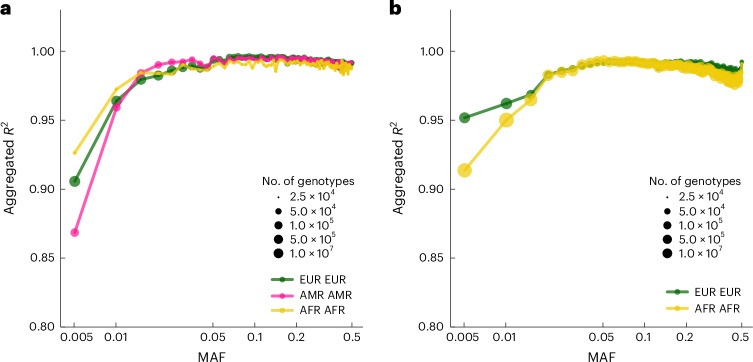
Imputation accuracy differentiates by local ancestry background at low allele frequencies. **a**, Aggregate *R*^2^ in the Paisa cohort. **b**, Aggregate *R*^2^ in the GPC cohort. Number of genotypes reflects one value for each sample and each SNP. Heterozygous ancestry results with nonreference concordance measurements are shown in Supplementary Fig. 8.

Aggregate *R*^2^ values stratified by local ancestry backgrounds across MAFs are shown in Fig. 5. For common variants (MAF ≥ 1%), imputation accuracy is qualitatively similar among various ancestral backgrounds, achieving >90% accuracy in both aggregate *R*^2^ as well as nonreference concordance (Supplementary Fig. 8a,b). Aggregated *R*^2^ and nonreference concordance for heterozygous diploid ancestry genotypes are shown in Supplementary Fig. 8c,d for the Paisa cohort. For less common SNPs, AMR ancestry has lower *R*^2^ than EUR and AFR ancestry within the Paisa cohort, a finding consistent with previous local ancestry imputation accuracy measurements^21^. Similarly, AFR ancestry generally does not perform as well as EUR ancestry within the GPC cohort, as expected. Accuracy differences among rare SNPs are small, with differences in aggregate *R*^2^ of less than 10%. Overall, these results suggest that low-coverage genotypes from the BGE platform can be statistically imputed with high accuracy, even when samples exhibit diverse continental admixture.

### BGE provides a cost-effective balance of variants

To summarize the balance of cost with variants assayed, we used a subset of NeuroGAP-Psychosis genomes (*N* = 79) with overlapping genetic data from deep WGS, BGE and Illumina GSA sites to count the number of variants assayed with imputation where relevant in Table 3 (Methods). We show that BGE captures the vast majority of common (94.4%) and rare (94.7%) coding variants found in 30× WGS, considerably more than can be imputed with GWAS arrays (66.1% and 27.6%, respectively). In the noncoding regions, more comparable fractions were imputed in BGE versus Illumina GSA for common (79.8% versus 67.6%) and rare variants (61.2% versus 24.5%); consistently more variants were imputed with BGE.

**Table 3:** BGE provides a cost-effective balance of variants accurately assayed compared with other data generation strategies

Variant frequency	Variant type	BGE	Illumina GSA (imputed)	30× WGS
MAF < 0.05	Noncoding SNPs	10,979,302 (imputed)	4,402,153	17,934,116
MAF ≥ 0.05	Noncoding SNPs	7,656,832 (imputed)	6,489,217	9,595,155
MAF < 0.05	Coding SNPs	140,310	40,863	148,090
MAF ≥ 0.05	Coding SNPs	55,373	38,756	58,630
Cost per sample (relative to WGS)		US$99 (0.28×)	US$65^a^ (0.19×)	US$350 (1×)

^a^Bulk pricing available for GSA with a minimum of 1,000 samples.

Monomorphic variants were removed, and variants were filtered for those with dosage *R*^2^ or INFO-score ≥0.8 (see more details in the Methods). Coding and noncoding regions are defined by capture region boundaries for BGE. Costs for BGE and WGS are starting costs described by https://broadclinicallabs.org/ as of 17 August 2024. Notably, these costs are basic lab processing costs only that do not include DNA extraction, project management, data storage, analytical or other costs.

## Discussion

The BGE sequencing approach represents an important advance by capturing genetic variation in an unbiased, high-quality and cost-effective manner. The development and testing of BGE as well as the in-depth analyses here clearly demonstrated that BGE sequencing can be accurately and efficiently scaled to empower human genetics research, especially in diverse and underrepresented populations. By unifying whole-genome common variants and coding rare variant analyses from a single sequencing run, our analyses showcase the potential of BGE to enhance genomic discoveries and improve variant detection. In contrast to a recent study that also combined low-coverage WGS with high-coverage WES^8^, blending library preparations together at optimized ratios before sequencing is unique to the BGE workflow. Our strategy balances variant calling and imputation accuracy at a lower sequencing costs (28% of WGS costs here, more expensive and variable previously). Furthermore, our approach has streamlined workflows, minimizing disjoint sample failures between WGS and WES data, thereby ensuring complete datasets for every successful sample sequenced. We see no clear limitations or biases introduced as a function of blended sequencing, as both deep exome variant identification and low-pass genome imputation show strong concordance when compared against independently sequenced callsets. However, we note that the choice of reference panel when performing imputation of low-pass genome sequencing data can introduce biases with respect to imputation accuracy. For example, we found lower aggregate *R*^2^ in AMR haplotypes from the Paisa population owing to fewer Indigenous American haplotypes in admixed AMR individuals relative to EUR and AFR ancestry individuals included in the HGDP+1kGP reference panel. Relatedly, while imputation panels that support GWAS arrays have increased in scale and diversity far beyond HGDP+1kGP, low-coverage imputation requires individual-level haplotype data and uses methods not currently supported by existing servers^16^. Relying solely on low-pass WGS would limit power to detect rare coding variants, as deep exome sequencing provides substantially better coverage. This higher coverage is crucial for identifying rare and ultrarare deleterious variants that often have larger and more interpretable effects^23,24^. By contrast, low-pass WGS and imputation often miss these variants owing to insufficient read depth, limiting the ability to confidently distinguish true variants from noise.

The BGE technology delivers consistent coverage and quality across cohorts, with high accuracy in calling coding CNVs and only minor differences in coverage based on saliva versus blood-based collection. In future saliva-based BGE sequencing, the protocol can be altered to draw a slightly larger aliquot in saliva samples to overcome this discrepancy. We find high recall and PPV when calling coding CNVs, with no discernable impact of saliva versus blood collection on CNV call rates. The slightly lower recall in calling coding CNVs overlapping <5 exons is due to the lower BGE target deliverables (90% of exome target reaching 10× depth) compared with deep exome protocols performed at the Broad Institute (85% of exome target reaching 20× depth). These benchmarks of comparability between protein-coding CNVs with BGE versus WES demonstrate a unique strength of the technology at this price point regarding assayable variants. However, additional work is underway to further quantify the full breadth of genome-wide structural variants detectable with low-pass WGS read data from BGE. While segregating structural variants can be tagged to some extent by SNPs^11^, the lack of a structural variant imputation service limits widespread adoption, and most rare and de novo structural variants would never be detected with imputation.

The BGE technology excels in common variant imputation from low-pass WGS reads, demonstrating consistently high concordance with GWAS array data across diverse cohorts, and within specific local ancestry tracts from large recently admixed cohorts. As low-pass WGS data is not biased toward a specific set of selected variants, the performance should remain robust and flexible compared with GWAS arrays across all varieties of future imputation reference panels, making it a powerful long-term tool for common genetic variant association studies. The choice of reference panel is a critical factor in determining imputation accuracy, particularly when working with sequencing data from diverse populations. While larger reference panels such as TOPMed contain a greater number of haplotypes, access limitations and methodological differences in imputation approaches can pose challenges. In our study, we used the HGDP+1kGP reference panel owing to its open accessibility, making it compatible to use with low-coverage sequencing imputation methods. Future efforts should focus on developing methods that allow seamless integration of diverse reference panels while accounting for differences in sequencing depth and data processing requirements.

Although GWAS array data are the most cost-comparable alternative to BGE, we acknowledge that they are not a gold standard for benchmarking imputation accuracy owing to their limited coverage and occasional errors^25^. We aimed to retain only the highest-quality genotypes in the selected array SNPs, but discrepancies arising between BGE and array data cannot be attributed solely to noise from low-pass imputation of BGE data. BGE is a particularly appealing genetic data strategy because it has similar costs to a GWAS array with genomic coverage akin to an exome plus array with imputation; deep WGS, considered a gold standard, identifies only ~1% more single-variant and gene-based associations than exome plus array data, despite costing more than 3.5× as much as BGE^26^.

Our evaluation across a highly ancestrally diverse suite of cohorts establishes BGE sequencing as a technology that bridges the gap between cost, comprehensive coverage and data quality for large-scale genomic studies. Its high-quality exome data, reliable CNV calling and accurate whole-genome imputation across diverse populations make it a valuable tool for advancing genomic research. By enabling more inclusive studies, BGE technology has the potential to enhance our understanding of genetic variation and its implications for human health around the world.

## Methods

### Ethics approval and consent to participate

All cohorts involving human participants that were included in this research have Institutional Review Board (IRB) approval following a full review of IRB applications and study consent forms by appropriate ethics committees. Details on each of the participating cohorts and IRB approvals are listed below:

Hispanic ASD samples, IRB title: The Study of Novel Autism Genes, protocol no. 2012p001018, IRB of record: Mass General Brigham (previously known as PARTNERS), principal investigator on IRB: M. Daly.

Chinese samples, IRB title: Neuropsychiatric genetics of a Chinese population from Shanghai Province, protocol no. IRB17-1379, IRB of record: Harvard T.H. Chan School of Public Health (HSPH), principal investigator on IRB: B. Neale.

NeuroGAP-Psychosis:Ethiopia and South African samples, IRB title: Neuropsychiatric Genetics of African Populations – Psychosis (NeuroGAP-Psychosis), protocol no. IRB17- 0822, IRB of record: Harvard T. H. Chan School of Public Health (HSPH), principal investigator on IRB: K. Koenen.

Paisa and Genomic Psychiatry Cohort, IRB title: Molecular Profiling of Psychiatric Disease, protocol no. 2014p001342, IRB of record: Mass General Brigham (previously known as Partners Healthcare), principal investigator on IRB: B. Neale.

Inflammatory Bowel Disease samples, IRB title: The Broad Institute Study of Inflammatory Bowel Disease Genetics, protocol no. 2013P002634, IRB of record: Mass General Brigham (previously known as Partners Healthcare), principal investigator on IRB: R. Xavier.

All participants who joined the studies listed under the above IRBs consented to the de-identified use of their data in disease research studies. During the consenting process, study staff explain the research goals and the basics of genetic processing activities. Participants are also given the option to opt out of studies if they choose. Samples used in this research and method development are all de-identified and have all been fully consented for inclusion in the study and any resulting publications.

### Wet lab protocol

#### Biological samples

BGE DNA samples have been extracted from saliva and whole blood specimens outside our laboratory. Samples derived from whole blood are generally preferred. Lower alignment rates of sequencing reads have been observed in saliva samples due to the presence of bacterial species. Furthermore, saliva samples can be more difficult to accurately quantify due to their consistency and heterogeneity. Following DNA extraction, we follow the BGE protocol described previously and shown in Supplementary Fig. 1.

#### PCR-free library preparation

After an initial quantification, DNA is normalized to 50 ng μl^−1^ and transferred into a 384-well plate. Normalized DNA is purified using an automated 2.75× solid-phase reversible immobilization (SPRI) clean-up with Ampure XP Agencourt beads (Beckman Coulter). Cleaned DNA is then quantified by spectrophotometry (Lunatic, Unchained Labs) and normalized to 25 ng μl^−1^. DNA (target of 134 ng input) undergoes a reduced and customized fragmentation/end-repair/A-tailing reaction for Illumina-compatible PCR-free library construction with custom NEBNext Ultra II FS DNA Library Preparation Kits (New England Biolabs) using the following conditions: 37 °C for 42.57 min, 65 °C for 30 min. Unique, dual-indexed adaptors (NEBNext Unique Dual Index UMI Adaptors, New England Biolabs) are ligated to fragments (20 °C for 20 min), and libraries undergo two consecutive SPRI size selections (0.5× and 0.55×). Cleaned and size-selected PCR-free libraries are quantified by qPCR (Kapa Library Quantification Kit, Roche), then normalized (to ~0.3–2 nM, depending on concentrations) before pooling into a single tube and concentrated (Unagi, Unchained Labs).

The oligonucleotides used are commercially available. From New England Biolabs, we use four plates containing 384 unique adapters (cat. no. E7874L, E7876L, E7878L and E7395L), which also include the forward and reverse primer mix for exome capture.

#### Exome-captured library preparation

An aliquot from the prenormalized and prepooled PCR free libraries is used as input for PCR amplification (cycles are 98 °C for 30 s; 12 cycles of 98 °C for 10 sec and 65 °C for 75 s; 65 °C for 5 min) using the NEBNext Ultra II FS Library Preparation Kit and primers from the indexed adaptor kit (New England Biolabs). PCR-amplified libraries are quantified by spectrophotometer (Lunatic), purified with a 1× SPRI-cleanup (Ampure) and normalized to 70 ng μl^−1^. Samples are then pooled and undergo exome capture (Twist Alliance Clinical Research Exome probes from Twist Biosciences) using the recommended hybridization-capture protocol for xGen Hybridization Capture Core Reagents (Integrated DNA Technologies).

#### Blending of PCR-free genome- and exome-captured libraries

PCR-free and exome-captured pools are both qPCR-quantified on the same qPCR run. Nanomolar concentrations are taken into account to calculate the appropriate volumes to blend 33% WES with 67% WGS. BGE samples are again qPCR-quantified for sequencer loading calculations.

#### Sequencing

BGE blended pools with 384 samples containing unique barcodes are sequenced across six lanes of NovaSeqS4 (Illumina) with 2× 150-bp runs.

### Datasets analyzed to evaluate BGE quality

BGE data used in these analyses were generated at the Broad Clinical Lab. Sample cohorts included in the dataset were recruited and submitted from the collaborating institutions for the PUMAS project, which includes cohorts from the GPC, Paisa population and NeuroGAP-Psychosis, as described below, in Table 2 and in Supplementary Table 1.

The GPC is a multi-institutional collaboration led by Rutgers University. The GPC resource includes a National Institute of Mental Health (NIMH)-managed repository of genomic samples at SAMPLED, genotypic and sequence data, and detailed clinical and demographic data for investigations of schizophrenia, bipolar disorder, obsessive–compulsive disorder and coronavirus disease from a variety of ancestries collected in the USA. Within this analysis, we included 4,553 samples with 3,926 passing QC filters.

The Paisa population is a genetic isolate from Colombia that has expanded rapidly following a series of migration-related bottlenecks. They have been the focus of genetics studies in neuropsychiatric disorders in the last decade. Paisa BGE data generated here emerged from a long-standing collaboration between teams at the Universidad de Antioquia, Medellin, Colombia, and University of California, Los Angeles, USA, to recruit samples from this population. Within the analysis, we included 9,007 samples, with 8,200 passing QC filters.

The Stanley Center at the Broad Institute of MIT and Harvard initiated the NeuroGAP-Psychosis project in 2015^27^, a collaboration with colleagues at Addis Ababa University in Ethiopia, KEMRI-Wellcome Trust in Kenya, Makerere University in Uganda, Moi University/Moi Teaching and Referral Hospital in Kenya, the University of Cape Town in South Africa, and the Harvard T. H. Chan School of Public Health in the USA. With initial plans to recruit 35,000 participants (half cases with a diagnosis of schizophrenia or bipolar disorder and half controls), the target was expanded to 39,000 participants via the PUMAS Project awarded by NIMH. Sample recruitment ultimately exceeded the revised target, with >42,000 samples collected across the five NeuroGAP-Psychosis collection sites. Within the analysis, we included 39,886 samples, with 35,138 passing QC filters.

The Australian Genetics of Bipolar Study^19^ led by the QIMR team is a national cohort of adults diagnosed with either type I or type II bipolar disorder. Genotypes were obtained using the Isohelix GeneFix GFX-02 2-ml saliva collection devices. The QSkin Sun and Health Study^20^ includes individuals from Queensland randomly sampled from the Australian Electoral Roll, with saliva samples for genotyping obtained using the Oragene DNA self-collection kit. Given the differing kits and enrollment strategies used in sample collection, we analyzed these cohorts separately for the imputation concordance analysis. Within the analysis and totaling from both QIMR cohorts, we included 7,499 samples, with 7,209 passing QC filters.

Data from the PUMAS project are being deposited at the NIMH Data Archive (NDA). At the time of manuscript submission, genomic data from the first 10,000 samples have been submitted. All remaining samples from the PUMAS grant will be submitted at the end of the grant period. Data that are designated with NDA-GRU data use will be deposited into DNA collection #3805. Data designated with Disease-Specific (Mental Health) – DS (Mental Health) will be deposited into DNA collection #4538. Finally, data designated Health/Medical/Biomedical, NDA-HMB-MDS will be deposited into DNA collection #4539.

### Exome quality filtering

We conducted QC filtering using Hail v0.28.128 to restrict to sites and variants with high confidence in exome data. We filtered out sites with more than six alleles that failed VQSR, were located in low-complexity regions, or fell outside the Twist target capture regions. Within an individual, genotype calls were filtered if read depth was <10×, genotype quality was <20, or allele balance was <0.2 or >0.8 in heterozygous calls, or <0.8 in homozygous alternate calls.

We filtered samples based on WES and WGS coverage, ancestry and WES sample quality metrics to restrict to high-quality samples for subsequent analysis. First, we removed samples with low WES or WGS coverage. Exome coverage per sample was determined by calculating the fraction of the exome target covered with at least 10× read depth. Samples with exome fractions less than 90% or estimated WGS coverage of less than 1× were removed. In addition, samples with chimeric or contamination read rates greater than 5% were removed, resulting in 853 removed samples.

Next, we determined genetic ancestry of the PUMAS samples using the quality-filtered WES portion of the BGE. We combined the PUMAS data with four reference panels with diverse ancestries: HGDP, 1kGP, AWI-GEN and the African Genome Variation Project (AGVP)^18,28,29^. Throughout this Article, we use ancestry labels assigned by existing genomic reference panels, including EUR (European), AFR (African) and AMR (Admixed American—an imprecise label introduced by the 1000 Genomes Project to describe individuals with recent admixture from multiple continents including Indigenous American ancestry). Sites overlapping between the PUMAS data and all reference datasets were filtered to biallelic variants with call rates >0.98 and MAFs >0.1%. We conducted linkage disequilibrium (LD) pruning to extract independent markers and calculated the top ten principal components across all samples using Hail’s hwe_normalized_pca function. We fit a random forest algorithm using the top ten principal components to the reference datasets of known ancestry and then applied the algorithm to the PUMAS data. We required PUMAS samples to have a probability of at least 0.7 to assign ancestry. Samples with probabilities less than 0.7 were removed (*N* removed = 1,799).

We calculated sample quality metrics using Hail’s sample_qc function. Within each ancestry and cohort combination, we removed samples with outlier values defined as values more than four median absolute deviations from the mean for the following metrics: *N* singletons, *N* insertions, *N* deletions, *N* transitions, *N* transversions, heterozygosity ratio, transition-to-transversion ratio and insertion-to-deletion ratio. Sample quality metrics filtering removed 2,317 samples.

Finally, we removed samples with discrepancies between imputed genetic sex and reported gender. Genetic sex was imputed separately for each genetic ancestry group by calculating the inbreeding coefficient on the X chromosome using common, independent markers after removing markers within the pseudoautosomal region. Samples with an inbreeding coefficient <0.6 were labeled ‘female’, and samples with an inbreeding coefficient >0.6 were called ‘male’. We removed samples whose imputed sex and reported gender did not match, resulting in 316 removed samples.

### CNV calling and QC

#### CNV calling

To call CNVs from the deep-coverage exome data, GATK-gCNV 4.1.0.0 was used on the exome intervals following a previously described pipeline^10^. GATK-gCNV adjusts for known WES read-depth confounders such as GC content and mappability, while simultaneously adjusting for unspecified technical batch confounders such as sample extraction, sequencing, library preparation and mapping quality. We took the read-depth data as input from the BGE samples over a set of canonical transcript genomic intervals. In brief, the raw sequencing files were compressed into counts of the reads over the set of annotated exons and used as input into GATK-gCNV. A principal component analysis-based approach was used on the compressed observed counts to identify differences in the capture kit. Within each cohort, principal component analysis was used to define ancestrally similar batches of 1,000 samples; from each, a random subset of 200 samples were used to train a CNV-discovery model tailored to each batch. Subsequently, a distance-based and hybrid-density-based clustering approach was used to curate samples into batches to process in parallel. Once batching determination was completed, GATK-GCNV was used on each batch and metrics for filtering were produced by the Bayesian model underlying the data to balance recall and PPV.

#### CNV benchmarking

To benchmark BGE CNV exome data, we used data from 100 quads from the SSC^30–32^, sequenced previously. Benchmarking was carried out on all rare CNVs (frequency <1%). The 100 quads have existing CNV calls^31,32^ from deep WGS (~30×), and the CNV calls from WGS were used as a gold standard to benchmark the BGE CNVs. Recall was calculated by the proportion of WGS data sites that had a match in the BGE CNV callset. Moreover, for any given site, if at least 50% of samples that had that variant in the WGS data also had a GATK-gCNV call with consistent directionality (duplication or deletion) that overlapped at least 50% of captured intervals, this was considered a correct call. The optimal recall and PPV were observed for CNVs spanning more than four exons in the 100 quads. Samples were removed if there were more than ten rare CNVs, defined as <1% frequency across the cohort. Any samples with >more than ten high-quality CNVs were removed. We additionally removed samples that failed SNV QC. Subsequently, CNVs were filtered to have a quality score >200 and restricted to <1% frequency for each ancestry. We further benchmarked the performance of de novo CNVs derived from BGE data using GATK-gCNV as a function of the number of captured exons of canonical transcripts compared with validated WGS de novo CNVs from the quads. We additionally benchmarked the CNVs in saliva and blood against two independent constrained gene sets (top 1,000) based on LOEUF^12^ from gnomAD v2 and GISMO-mis^13^.

#### Structural variant calling

The structural variants were identified using VISTA v1^14^ for deletion and INSurVeyor v1.1.3^15^ for insertions. VISTA is an ensemble tool that combines the best predictions from multiple individual callers, including Manta, Delly and others. We ran the individual callers and combined the results while applying the default parameters and quality filters. For InSurVeyor, we also applied the default parameters, which produced a high-quality callset that was used for analysis.

### Imputation

To impute the low-coverage WGS data, we used the Genotype Likelihoods IMputation and Phasing (GLIMPSE2 v2.0.0) method^16^. We used the HGDP+1kGP reference panel^18^ after filtering out singleton variants and indels, resulting in roughly about over 67 million variants available to be imputed.

To phase and impute this large-scale data in a cost-effective manner, we used Broad’s Hail Batch Service to submit randomized batches of 200 individuals. Then, we merged imputed results from the batches using bcftools. In total, we imputed over 55,000 samples for just under US$20,000, averaging about US$0.36 per sample (Supplementary Table 5).

AFs were corrected after merging by calculating a weighted mean of the batch allele frequencies (AFs) based on individual batch values (for *i* batches and *N* samples per batch):

$${\mathrm{AF}}_{\mathrm{cohort}}=\frac{\sum {\mathrm{AF}}_{i}{N}_{i}}{{\sum N}_{i}\,}.$$

Similarly, INFO scores (*I*) were corrected post-merge via

$${I}_{\mathrm{cohort}}=1\,-\,\frac{\sum (1-{I}_{i})\times 2{N}_{i}\,\times \,{\mathrm{AF}}_{i}(1-{\mathrm{AF}}_{i})}{2\times \sum {N}_{i}\times \frac{\sum {\mathrm{AF}}_{i}{N}_{i}}{\sum {N}_{i}}\left(1-\frac{\sum {\mathrm{AF}}_{i}{N}_{i}}{\sum {N}_{i}}\right)}.$$

This process resulted in a final set of chromosome-specific BCF files, with all individuals of a cohort included and allele frequencies and INFO scores updated to reflect the full sample size.

### QC of GSA data

Variants genotyped via the Illumina GSA platform were first filtered for a SNP call rate of at least 95%; then samples were filtered to have a call rate of at least 98%, heterogeneity *F* statistic or inbreeding coefficient |*F*_HET_| <0.20, and concordance between genetically inferred and reported sex. A second SNP filtering step with a call rate of at least 98% was performed after the sample QC, followed by filtering steps to remove SNPs with missingness differences >2% between cases and controls, and Hardy–Weinberg equilibrium *P* value of 1 × 10^−6^ for controls and 1 × 10^−10^ for cases. We used King^33^ to calculate the genetic relationship matrix and removed one sample in any pairs of individuals with second-degree or closer relatives. Finally, we used the conform-gt tool provided by the Beagle V5.4 imputation software^34^ to align the strand and allele order within the GSA VCFs to the HGDP+1kGP reference panel^18^.

### Concordance between BGE and GSA GWAS array data

To evaluate BGE data accuracy post-imputation, we compared BGE with previously generated Illumina GSA data as ground truth on the subset of individuals with both data types (Supplementary Table 4). Imputed variants were filtered to match those in the GSA. We computed nonreference concordance and aggregated *R*^2^ as a function of MAF for each cohort. To evaluate nonreference concordance^3^, we define it as the number of true positives divided by the sum of the true positives, false negatives and false positives (excluding missing sites and SNPs with only reference alleles within the GSA subset), as shown in the formula below. We computed aggregated *R*^2^ per MAF bin by stacking all SNP dosages (from the imputed data) and genotype calls (from the GSA data) within a MAF bin, and calculating the squared Pearson’s correlation coefficient. Note that, to avoid discrepancies in MAF for the NeuroGAP-Psychosis subsets (each with around 160 individuals), MAF values were based on the HGDP+1kGP AFR subset rather than in-sample allele frequencies. For the larger datasets, allele frequency values were taken from in-sample estimates.

$$\begin{array}{l}{\mathrm{non-ref-concordance}}({{\mathrm{SNP}}}_{j})\\=\displaystyle\frac{{\rm{\#}}\,{\rm{true\; positives}}}{{\rm{\#}}\,{\rm{true\; positives}}+{\rm{\#}}\,{\rm{false\; positives}}+{\rm{\#}}\,{\rm{false\; negatives}}}.\end{array}$$

Let *X*_GSA_ be the matrix of genotypes for a given set of individuals and SNPs based on GSA array data, while *X*_BGE, imputed_ is the matrix of imputed dosages for the same set of SNPs and individuals. We can calculate aggregated *R*^2^ per a given MAF bin for a SNP as follows^35^:

$$\begin{array}{l}\mathrm{Aggregate}\,{R}^{2}\,\mathrm{per}\,\mathrm{MAF}\,\mathrm{bin}\\={\left({\mathrm{Pearson}}^{^{\prime} }{\rm{s}}\;\mathrm{correlation}\left(\mathrm{vec}\left({X}_{\mathrm{GSA}}\right),\mathrm{vec}\left({X}_{\mathrm{BGE},\mathrm{imputed}}\right)\right)\right)}^{2}.\end{array}$$

### Concordance between BGE and GSA by local ancestry inference

We also evaluated post-imputation accuracy in BGE data stratified by each genotype’s local ancestry background. We focus specifically on the Paisa and GPC populations, which have seen recent admixture between European, Native American Indigenous American and African continental ancestry. We first phased array genotypes via Beagle 5.4^36^ using the full HGDP+1kGP reference panel (*n* = 4,099)^18^. Next, we followed the Tractor^37^ tutorial and computed local ancestry with RFMix2 software^22^. For the Paisa cohort, we modeled three-way local ancestry to capture admixture represented by EUR, AFR and AMR (as a proxy for Indigenous American ancestry) populations in the HGDP+1kGP reference panel. Specifically, we filtered the reference panel to only samples with AFR/EUR/AMR population labels. For local ancestry inference to be most useful, reference samples themselves should be representative of ancestral populations and non-admixed. Because AMR samples tend to themselves be recently admixed, we excluded 358/549 reference AMR samples that have <90% Indigenous American ancestry as measured by RFMix2. As a result, the total number of reference samples *N*_total _= 1,935 are split among 191 AMR, 752 EUR and 992 AFR. For the GPC population, we inferred two-way local ancestry to capture European and African admixture. When running RFMix2, we used the optional EM flag set to one iteration to account for residual admixture within the subsetted reference panel. Finally, we also included the ‘-n 5’ flag to account for reference panel sample size imbalance.

After local ancestry inference, two metrics were used to evaluate imputation accuracy for different ancestry backgrounds, including aggregate *R*^2^ and aggregate nonreference concordance, computed by stacking all SNPs within a MAF bin and evaluating a single nonreference concordance value. This is analogous to the aggregate *R*^2^ formula except that we compute concordance rather than squared correlation after stacking SNPs column-wise. Note that we favor the aggregate nonreference concordance metric as opposed to averaging SNP-by-SNP concordances due to the presence of rare SNPs. This is because a rare SNP may have only several copies of the minor allele, and by chance, they may all reside on one specific ancestry background. This leaves no copies of minor alleles for other ancestry backgrounds. Averaging SNP-by-SNP nonreference concordances will therefore include excess zeros for the remaining ancestries, artificially deflating their accuracy. Stacking rare SNPs rescues concordance for rare SNPs among all ancestry backgrounds by virtue of including more copies of the minor allele, ultimately producing more accurate and interpretable results.

### Number of variants per data generation type

We evaluated a subset of samples from the NeuroGAP-Psychosis data (*N* = 79) that were assayed across three different data generation strategies, including BGE, Illumina GSA arrays and 30× WGS. To count the number of variants per MAF category in the noncoding BGE data, we used the imputed data imputed with GLIMPSE2 after filtering for an INFO score of at least 0.8 and removing monomorphic variants. For the coding regions, we utilized the high-coverage exome data rather than the imputed data, filtering for VQSR PASS variants as well as excluding locus control regions and filtering for monomorphic variants. We required genotypes to have genotype quality >50 and depth >13, and normalized Phred-scaled likelihoods to be >50. These same filters were applied for both coding and noncoding regions in the WGS dataset. For the Illumina GSA GWAS array data, we used the imputed data generated previously^3^ using the phase 3 1000 Genomes Project reference panel with BEAGLE v5.1 for all categories, filtering for dosage *R*^2^ of at least 0.8 and removing monomorphic variants. Coding regions were defined by Twist target capture region intervals.

### Reporting summary

Further information on research design is available in the Nature Portfolio Reporting Summary linked to this article.

## Online content

Any methods, additional references, Nature Portfolio reporting summaries, source data, extended data, supplementary information, acknowledgements, peer review information; details of author contributions and competing interests; and statements of data and code availability are available at 10.1038/s41588-026-02669-w.

## Supplementary information

Supplementary Information Supplementary Methods, Supplementary Tables 1–5, Supplementary Figs. 1–8 and Supplementary References.

Reporting Summary

## Data Availability

We describe all datasets within this Article or Supplementary Tables 1 and 2. Genome Reference Consortium Human Build 38 can be accessed at https://www.ncbi.nlm.nih.gov/assembly/GCF_000001405.40/. Data generated and analyzed come from several studies, including dbGaP study accessions phs001642.v1.p1 and phs002502.v3.p1. The NeuroGAP cohort data have been uploaded into the NIMH Data Archive (NDA) and are located in study accessions #3805 (NDA-GRU), #4538 (Disease-Specific (Mental Health)) and #4539 (NDA-HMB-MDS). There is currently an embargo on access to this data via NDA until July 2027. The NeuroGAP cohort established this embargo while the BGE sequence technology was under development and data generation timelines were uncertain. The NeuroGAP cohort utilized the H3Africa framework described previously^38^, setting an 11-month publication embargo from the end of NIMH funding (U01MH125047), and this embargo was agreed upon by all NeuroGAP PIs, their institutions and NIMH. For questions about accessing the NeuroGAP dataset before it is available in the NDA, please contact R.E.S. at rstroud@hsph.harvard.edu or the NeuroGAP administrative team at neurogapadmin@hsph.harvard.edu. Sequence data available for each study accession include a CRAM file per individual as well as quality-controlled genomic variant data calls (vcf files) that are compatible with common software tools such as hail or bcftools. For questions about accessing the QIMR dataset please contact S.E.M. at sarah.medland@qimrb.edu.au.

## Code availability

Code for conducting exome QC, imputation and concordance analyses is available via GitHub at https://github.com/atgu/bge_analysis or via Zenodo at 10.5281/zenodo.13785533 (ref. ^39^). Note that there is also a wiki page in this repository that describes how to perform common genetic analyses. Code for running the gCNV pipeline is available via GitHub at https://github.com/broadinstitute/gatk/tree/4.1.0.0/scripts/cnv_wdl/germline.
