# Supplementary material for: A blended genome and exome sequencing method captures genetic variation in an unbiased and cost-effective manner
Source: Nat Genet. 2026 Jul 8;58(8):1813–21. doi: 10.1038/s41588-026-02669-w (PMC13447102; doi:10.1038/s41588-026-02669-w)
Supplement: Supplementary file 1 [file 41588_2026_2669_MOESM1_ESM.pdf]

nature genetics

Supplementary information

https://doi.org/10.1038/s41588-026-02669-w

A blended genome and exome sequencing method captures genetic variation in an unbiased and cost-effective manner

In the format provided by the authors and unedited

Supplementary Methods

Technical development of the BGE protocol

We ran multiple experiments to determine the best nanomolar blending ratio of whole exome to whole genome library and the amount of sequencing required per sample. Through iterative tests on samples derived from Hispanic patients[1], we titrated both the WES:WGS blending ratio and sequencing coverage to determine the best conditions for calling both coding WES and common WGS variants. Our objective was to achieve >99% r2 imputation concordance of BGE data to 30x whole genome data. We wanted to remain cost-efficient in sequencing, with a low coverage genome to impute the common variants, while providing enough exome coverage for 90% of exome bases to reach 10x depth. We tested, in this order, 67% WES:33% WGS for 96 samples per lane of NovaSeqS4 (Illumina, San Diego, CA, USA), 67% WES:33% WGS for 48 samples/ lane, 60% WES:40% WGS for 48 samples/ lane, 40% WES60% WGS for 48 samples/ lane, and 33% WES:67% WGS for 64 samples/ lane.

In order to analyze R^2 imputation concordance, there were 31-62 samples among these blending ratio test samples for which we had deep whole genome data (mean 30x coverage) that could serve as a comparison truth set for concordance analysis. (Some samples were subsequently excluded during these blending ratio testing iterations as they were eventually depleted of raw DNA material for sequencing). For both BGE CRAMs and a selection of SNPs from the deep whole genomes that are present in the Infinium Global Screening Array (GSAv1.0), we used the Haplotype Reference Consortium[2] for phasing and imputation. Imputed calls from selected GSA SNPs were performed in RICOPILI[3] which uses Eagle 2.3.5 for phasing and Minimac3 for imputation. We ran the BGE CRAMs through GLIMPSE[4] version 1.1.1 for genotype refinement, phasing, and imputation. Before testing R^2 concordance of the imputed SNPs to the deep whole genomes, we first restricted our deep genome calls only to GATK PASSing variants. Among each imputed callset, we filtered our calls to a higher quality subset of imputed calls (INFO score > 0.8 and imputed MAF difference < 15% to whole genome MAF). Per-sample concordance was then tested on all selected genotypes present in both callsets, with concordance being the squared correlation coefficient after confirming the match of locus, reference, and alternate alleles in both datasets.

The first test condition (67% WES:33% WGS and 96 samples per lane of NovaSeqS4) resulted in 29x WES and 1.5x WGS coverage per sample and >99% R^2 imputation concordance of coding variants, but < 99% for whole genome variants. We then continued to iteratively test the other blending and sequencing coverage conditions. After plotting the varying levels of R^2 imputation concordance (**Supplementary Figure 1a,b**), we determined that a blend of 33% WES and 67% WGS for 64 samples/ lane provided adequate coverage of each (2-3x WGS and 30-40x WES per sample) for calling common WGS and coding WES variants (MAF > 5%), with >99% r2 imputation concordance to 30x WGS data for both. BGE data also provided higher r2 imputation concordance to 30x WGS data than imputation from selected SNPs in the Infinium Global Screening Array (Illumina) pulled from 30X WGS.

Using the optimized blending and sequencing coverage condition (33% WES:67% WGS for 64 samples/lane), we next piloted the full BGE process on a larger 836 sample set from blood (452) and saliva-derived (384) gDNA. These samples were derived from 64 South African, 320 Ethiopian (both from the Neuropsychiatric Genetics of African Populations - Psychosis Study and sequenced under the NIMH Populations Underrepresented in Mental illness Association Study (PUMAS)), 384 Chinese (Neuropsychiatric genetics of a Chinese population from Shanghai Province) patients, as well as 68 from the Broad Institute Study of Inflammatory Bowel Disease Genetics. Input gDNA was normalized to 50 ng/uL and plated into a 384-well PCR plate. We purified the normalized gDNA using a 2.75X solid phase reversible immobilization (SPRI) clean-up with Ampure beads (Beckman Coulter, Indianapolis, IN, USA). Purified gDNA was then quantified by spectrophotometry and re-normalized to 25 ng/uL. A target of 134 ng DNA underwent a customized fragmentation/end-repair/ A-tailing reaction for Illumina-compatible PCR-free library construction with NEBNext Ultra II FS DNA Library Preparation Kits (New England Biolabs, Ipswich MA, USA) using the following conditions: 37℃ 42.57 min, 65℃ 30 min. Next, we ligated unique, dual-indexed adaptors (NEBNext Unique Dual Index UMI Adaptors, New England Biolabs) to fragments (20℃ 20 min) and libraries underwent two consecutive SPRI automated size-selections. Libraries were then qPCR-quantified (Kapa Library Quantification Kit, Roche, Basel, Switzerland), normalized before pooling into a single tube, and concentrated. Pooling, size-selection, and library construction were carried out in batches of 192 samples, with one CRAM file produced per sample.

The 836 pilot samples yielded a mean of 2.6x estimated WGS coverage and 34.1x mean WES coverage per sample. An average of 96.35% of exome bases were covered at 10x. Mean percentage of all reads aligned to the reference was 97.14 (99.5% for blood and 94.78% for saliva, with a low of 72.9% for saliva due to bacterial DNA). Twenty two African saliva samples from this pilot study had previously been whole-genome sequenced (30x), allowing us to perform the same concordance analysis as described in the earlier experiments (using the 1000 Genomes Project reference panel[5]). We used the same filtering parameters as above, with an additional filter for genotypes with posterior probabilities < 0.9, which had a negligible impact on genotyping call rates but did increase our concordance by a few percentage points. For the larger 836 pilot sample set, we stratified our concordance analysis by allele frequency bins to see how lower frequency SNPs were performing. Allele frequencies were estimated from 371 samples of African ancestry in the pilot cohort (64 ascertained from the University of Cape Town, South Africa, and 371 ascertained from Addis Ababa University, Ethiopia). Singletons are variants with only a single alternate allele genotype present in the 371 samples, and MAF < 1% and MAF < 5% both include singletons in their frequency bin (**Supplementary Figure 1c**). In agreement with our blending optimization experiments, we observed >99% R^2 imputation concordance of BGE data with 30x whole genome data, maintained high concordance over the exome, and maintained concordance levels well above 90% among singleton SNPs for these 22 samples (**Supplementary Figure 1d**), demonstrating the repeatability and utility of the method.

Supplementary Tables

Supplementary Table 1. Cohort breakdown of data included in these analyses with phenotype and recruitment information.

The first institution listed in the "Submitted by" column was responsible for recruiting participants; the second institution was the US-based NIH prime institution. Phenotypes are: SCZ=schizophrenia, BP=bipolar, MDD=major depressive disorder. # Psychosis cases refer to instances that do not fit other diagnoses. Quality control includes filtering on WES and WGS coverage, genetic ancestry, outlier filtering on sample quality metrics, and checking for discrepancies between genetic sex and reported gender.

**Cohort name**	**Country of Origin**	**Tissue**	**Submitted by**	**# Total Pre-QC**	**# SCZ Cases**	**# BP Cases**	**# MDD Cases**	**# Psychosis Cases**	**# Controls**	**# Total Post-QC**
NeuroGAP - Addis Ababa University	Ethiopia	Saliva	AAU/ HSPH	11,715	4,171	1,209	0	16	5,631	11,027
NeuroGAP - KEMRI	Kenya	Saliva	KEMRI/ HSPH	3,078	729	485	0	407	1,268	2,889
NeuroGAP - Moi Teaching and Referral Hospital	Kenya	Saliva	Moi/ HSPH	5,040	1,385	968	0	2	2,361	4,716
NeuroGAP - University of Cape Town	South Africa	Saliva	UCT/ HSPH	8,747	2,015	710	0	34	3,020	5,779
NeuroGAP - Makerere University	Uganda	Saliva	Makerere Uni/ HSPH	11,306	1,501	2,784	1	955	5,485	10,727
Paisa	Colombia	Blood	UdeA/ UCLA	9,007	1,369	2,984	2,539	22	1,284	8,200
Genomic Psychiatry Cohort (GPC)	USA	Blood	Rutgers	4,553	906	865	30	2	1,865	3,926
			**Total**	**53,446**	12,076	10,005	2,570	1,438	20,914	**47,264**

Supplementary Table 2. Ancestry composition of each cohort. We used a random forest trained using the first 10 principal components on four reference panels: the harmonized HGDP+1KG dataset[6], AGVP[7], and AWI-Gen[8]. We required a random forest probability > 0.7 to assign ancestry labels. Sample sizes show pre-QC / post-QC values, as ancestry outlier filtering based on principal components centroid filtering was a QC criteria.

**Cohort Name**	**AFR**	**AMR**	**CSA**	**EAS**	**EUR**	**MID**	**OCE**
**NeuroGAP - Addis Ababa University**	11,219 / 11,075	0 / 0	0 / 0	0 / 0	0 / 0	37 / 8	0 / 0
**NeuroGAP - KEMRI**	3,054 / 2,890	1 / 0	5 / 4	0 / 0	0 / 0	12 / 8	0 / 0
**NeuroGAP - Moi Teaching and Referral Hospital**	4,804 / 4,784	0 / 0	0 / 0	0 / 0	0 / 0	0 / 0	0 / 0
**NeuroGAP - University of Cape Town**	7,501 / 5,723	99 / 2	611 / 200	5 / 0	230 / 179	6 / 0	3 / 0
**NeuroGAP - Makerere University**	10,934 / 10,819	0 / 0	1 / 0	0 / 0	0 / 0	0 / 0	0 / 0
**Paisa - Colombia**	61 / 14	8,878 / 8,198	0 / 0	1 / 0	31 / 12	1 / 0	0 / 0
**GPC - USA**	3,861 / 3,448	547 / 454	20 / 8	9 / 5	58 / 47	10 / 5	0 / 0

Supplementary Table 3. Number of imputed SNPs by minor allele frequency.

MAF bin	NeuroGAP unfiltered	NeuroGAP INFO>=0.8	GPC unfiltered	GPC INFO>=0.8	Paisa unfiltered	Paisa INFO>=0.8
**[0,0.01]**	52,969,838	15,808,286	53,368,842	24,003,218	58,363,478	23,364,001
**(0.01,0.02]**	3,381,317	3,337,758	2,986,989	2,972,371	1,775,145	1,766,482
**(0.02,0.03]**	1,681,557	1,670,725	1,599,805	1,594,889	733,280	731,040
**(0.03,0.04]**	1,084,382	1,079,477	1,069,749	1,067,513	452,302	451,298
**(0.04,0.05]**	790,740	788,116	793,965	792,804	352,733	352,164
**(0.05,0.1]**	2,249,890	2,244,708	2,319,863	2,318,137	1,177,405	1,176,502
**(0.1,0.15]**	1,247,955	1,246,018	1,271,570	1,271,093	816,420	816,179
**(0.15,0.2]**	876,932	875,664	890,479	890,207	669,883	669,746
**(0.2,0.25]**	667,141	666,200	669,093	668,921	572,871	572,800
**(0.25,0.3]**	548,769	548,037	539,499	539,384	514,843	514,792
**(0.3,0.35]**	480,545	479,879	475,999	475,875	472,541	472,504
**(0.35,0.4]**	433,172	432,526	431,230	431,144	458,325	458,288
**(0.4,0.45]**	406,113	405,543	403,343	403,235	434,695	434,664
**(0.45,0.5]**	398,198	397,559	396,123	396,019	422,628	422,584
**Total**	67,216,549	29,980,496	67,216,549	37,824,810	67,216,549	32,203,044

Supplementary Table 4. Number of overlapping samples per cohort with BGE and GSA array data.

Dataset	N samples with BGE and GSA data
NeuroGAP - AddisEthiopia	158
NeuroGAP - KEMRIKenya	183
NeuroGAP - MoiKenya	157
NeuroGAP - CapeTown South Africa	162
NeuroGAP - Makerere Uganda	178
Paisa - Colombia	1,191
GPC - USA	3,932
QIMR - AGBP	3,664
QIMR - QSkin	3,545

Supplementary Table 5. Costs of imputation per cohort.

Cohort	Sample Size	Cost	Cost Per Sample
**NeuroGAP**	35,279	$12,763.23	0.361
**Paisa**	8,317	$3,025.45	0.363
**GPC**	3,967	$1,406.46	0.354
**QIMR**	7,499	$2,732.96	0.364
**Total**	**55,062**	**$19,928.10**	**0.361**

Supplementary Figures

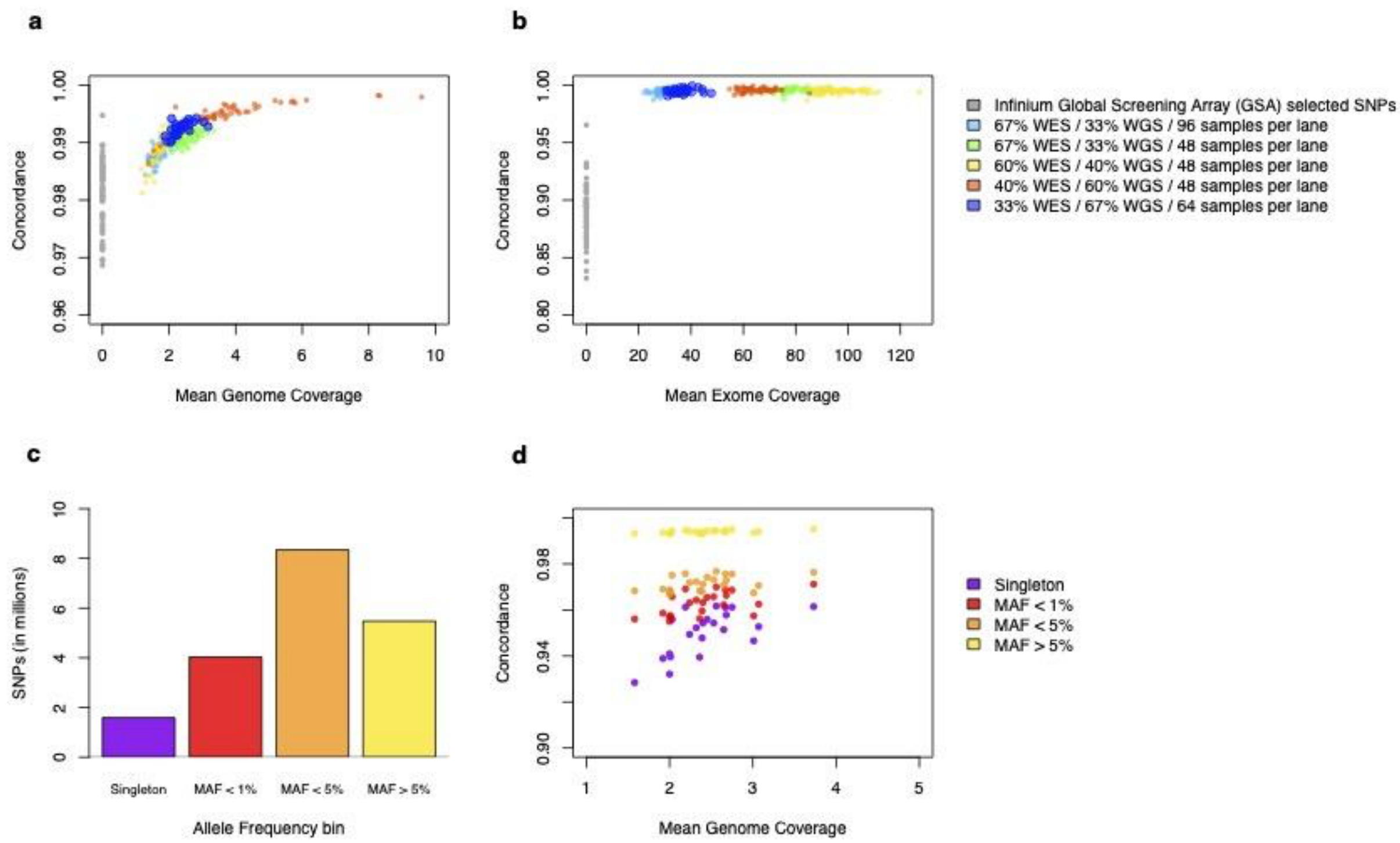

Supplementary Figure 1: Imputation genotype concordance with deep whole genome sequencing across experiments. a, Per-sample genotype concordance of filtered Haplotype Reference Consortium (HRC) imputed variants to deep whole genome variants (y-axis) as a function of mean coverage (x-axis) in the low-pass genome. Gray points are not using low pass genome data, but HRC imputation results from Infinium Global Screening Array (GSA) selected SNPs from 30x whole genome sequencing. The larger dots represent the selected blending proportion and lane throughput (33% Whole Exome Sequence / 67% Whole Genome Sequence / 64 samples per lane of NovaSeq S4) for the BGE product. **b,** Per-sample genotype concordance of filtered imputed variants restricted to annotated protein-coding variants with MAF < 5% in the deep whole genome sample set (y-axis) as a function of target mean coverage (x-axis) in the exome. **c,d,** SNP counts (c) and concordance (d) from imputation of the full BGE pilot cohort at various allele frequency bins. Imputation was performed using the 1000 Genomes Project reference panel, and allele frequencies were estimated from 371 samples of African ancestry in the pilot cohort. Singletons are variants with only a single alternate allele genotype present in the 371 samples. Per-sample genotype concordance was done on 22 African ancestry samples with deep whole genome data.

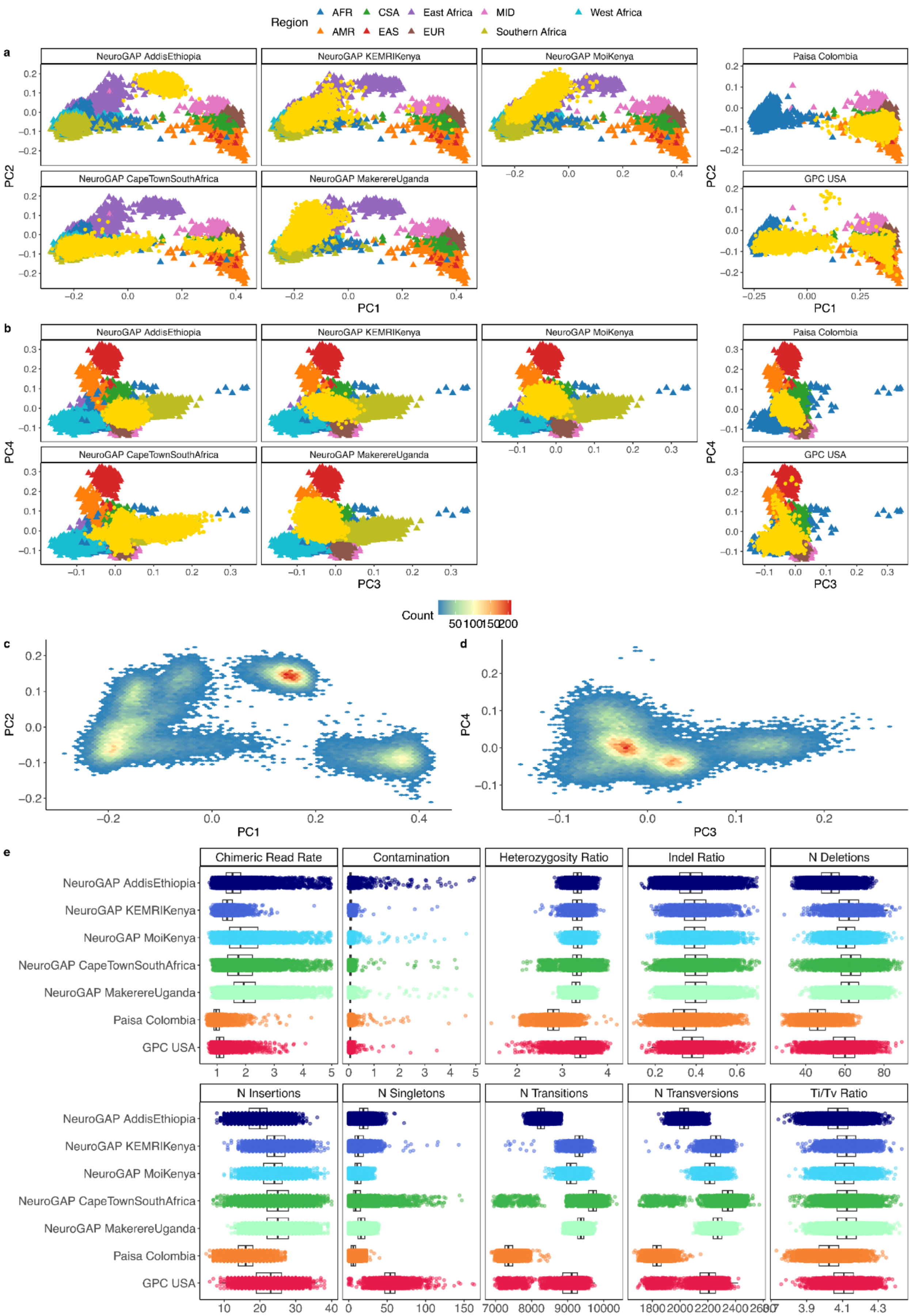

Region
AFR
AMR
CSA
EAS
East Africa
EUR
MID
Southern Africa
West Africa
a
b
c
d
e
NeuroGAP AddisEthiopia
NeuroGAP KEMRIKenya
NeuroGAP MoiKenya
NeuroGAP CapeTownSouthAfrica
NeuroGAP MakerereUganda
Paisa Colombia
GPC USA
PC1
PC2
PC3
PC4
Count
Chimeric Read Rate
Contamination
Heterozygosity Ratio
Indel Ratio
N Deletions
N Insertions
N Singletons
N Transitions
N Transversions
Ti/Tv Ratio

Supplementary Figure 2. Principal components with reference panels for all PUMAS cohorts and additional sample quality control metrics.

a. PC1 vs PC2 for PUMAS samples (yellow) and reference samples stratified by cohort. For NeuroGAP sites, reference samples are color-coded by region, including four reference panels with diverse ancestries: HGDP, 1KGP, AWI-GEN, and AGVP. For Paisa and GPC, reference panels were limited to HGDP and 1KGP.
b. PC2 vs PC3 for PUMAS samples (yellow) and reference samples stratified by cohort. For NeurGAP sites, reference samples are color-coded by region, including four reference panels with diverse ancestries: HGDP, 1KGP, AWI-GEN, and AGVP. For Paisa and GPC, reference panels were limited to HGDP and 1KGP.
c. Density plots of PC1 vs PC2 for all PUMAS samples.
d. Density plots of PC3 vs PC4 for all PUMAS samples.
e. Estimated chimeric read rates, contamination rates, heterozygosity ratio, indel ratio, number of deletions and insertions, number of singletons, number of transitions and transversions, and Ti/Tv (transition/transversion) ratio per sample per cohort.

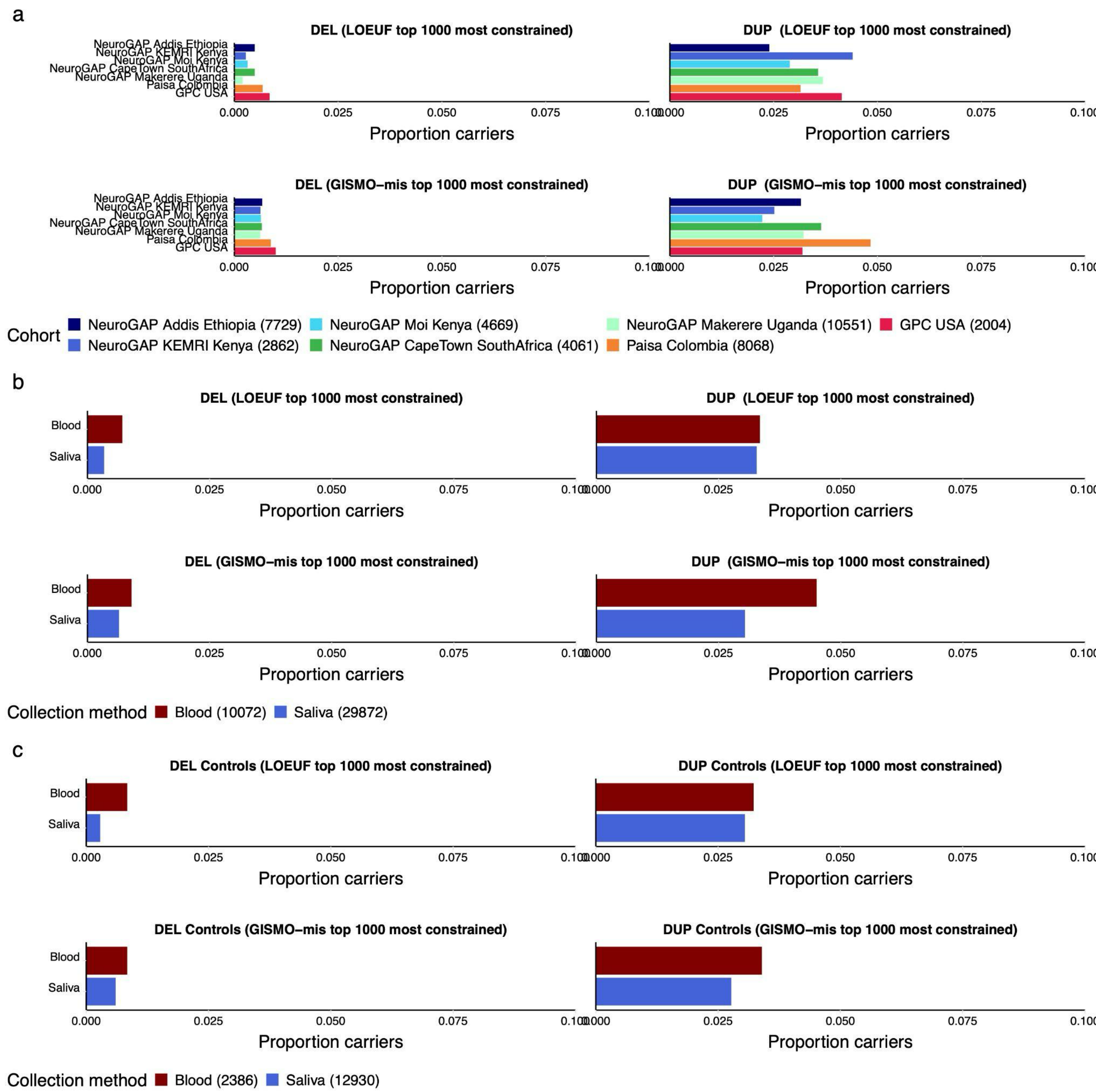

Supplementary Figure 3. Proportion of CNV carriers across the top 1000 most constrained genes for LOEUF and GISMO-mis.

a. Across all cohorts
b. Across collection method. A greater proportion of blood samples were carriers for CNVs compared to saliva samples (*OR = 1.1201, p = 0.0587*; *OR = 1.4974, p = 2.45e-13* for LOEUF and GISMO-mis, respectively)
c. Control samples across collection method. We observe a greater proportion of blood sample CNV carriers compared to saliva sample CNV carriers (*OR = 1.2001, p = 0.1243; OR = 1.2625, p = 0.0446* for LOEUF and GISMO-mis, respectively).

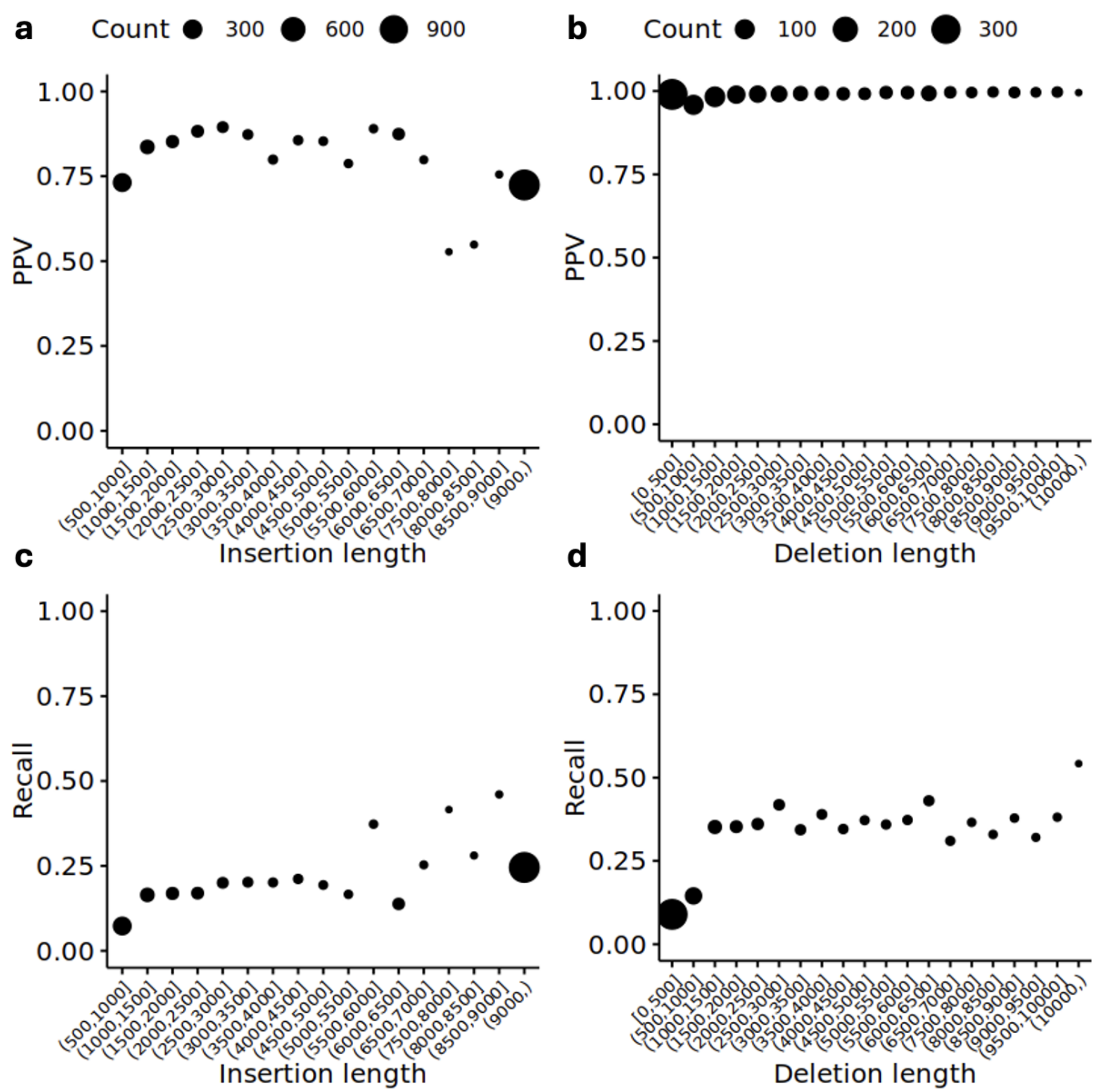

Supplementary Figure 4. Structural variants in the Simons Simplex Collection.

Positive predictive value (PPV) and recall of calling deletion and insertion events in the BGE data.

a. PPV of insertions,
b. PPV of deletions
c. Recall of insertions
d. Recall of deletions. For deletions, the scores were calculated for each length interval (500 bp) and events with length ≥ 10,000 bp were grouped into one level. Insertions were grouped into intervals spanning 5 bp. The size of the dots represented the number of events present.

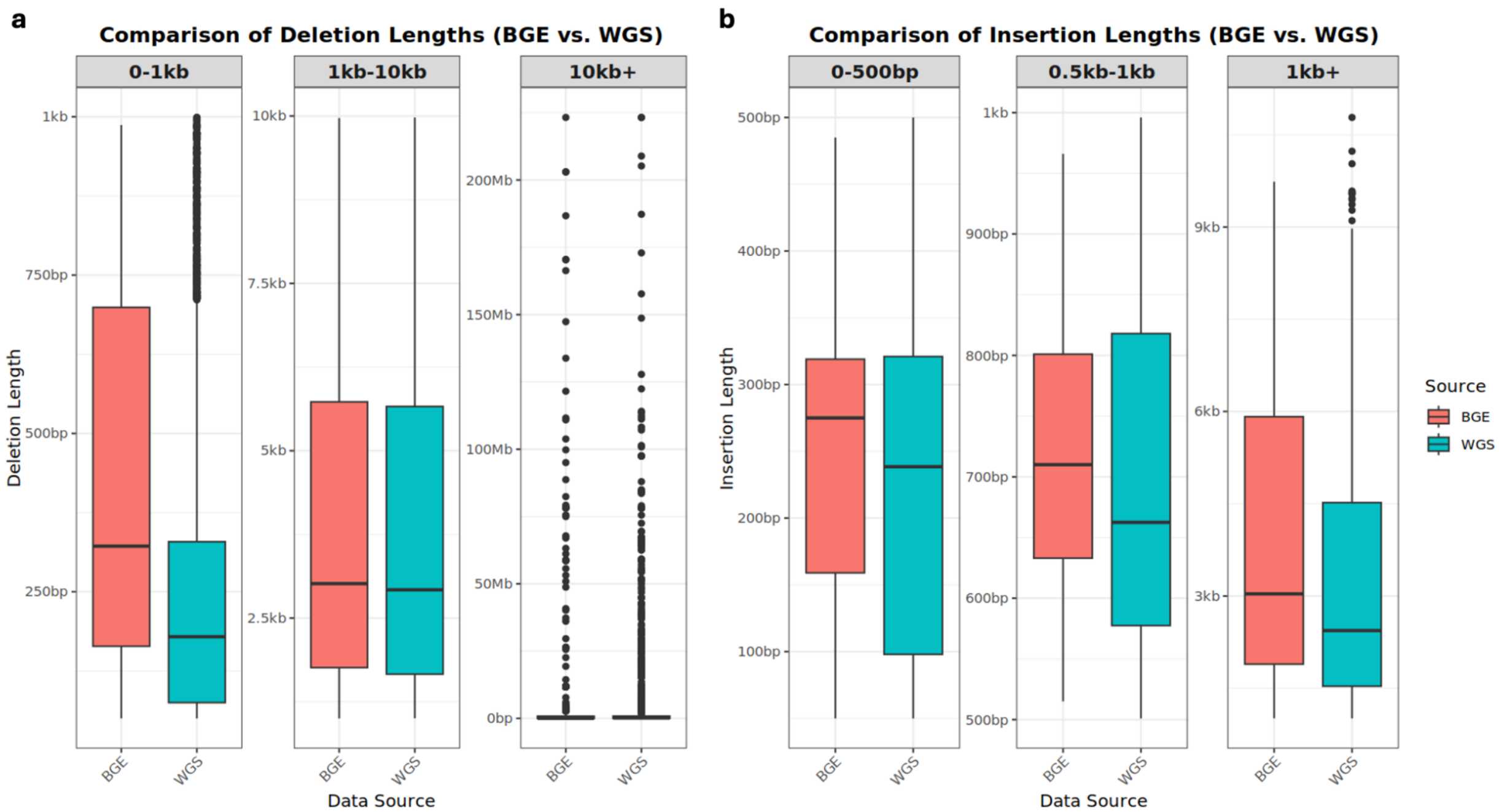

Supplementary Figure 5. Structural variant size ranges using BGE vs WGS in the Simons Simplex Collection.

a. Deletion length distributions using BGE and WGS for size ranges under 1kb, between 1-10kb, and over 10kb.
b. Insertion length distributions.

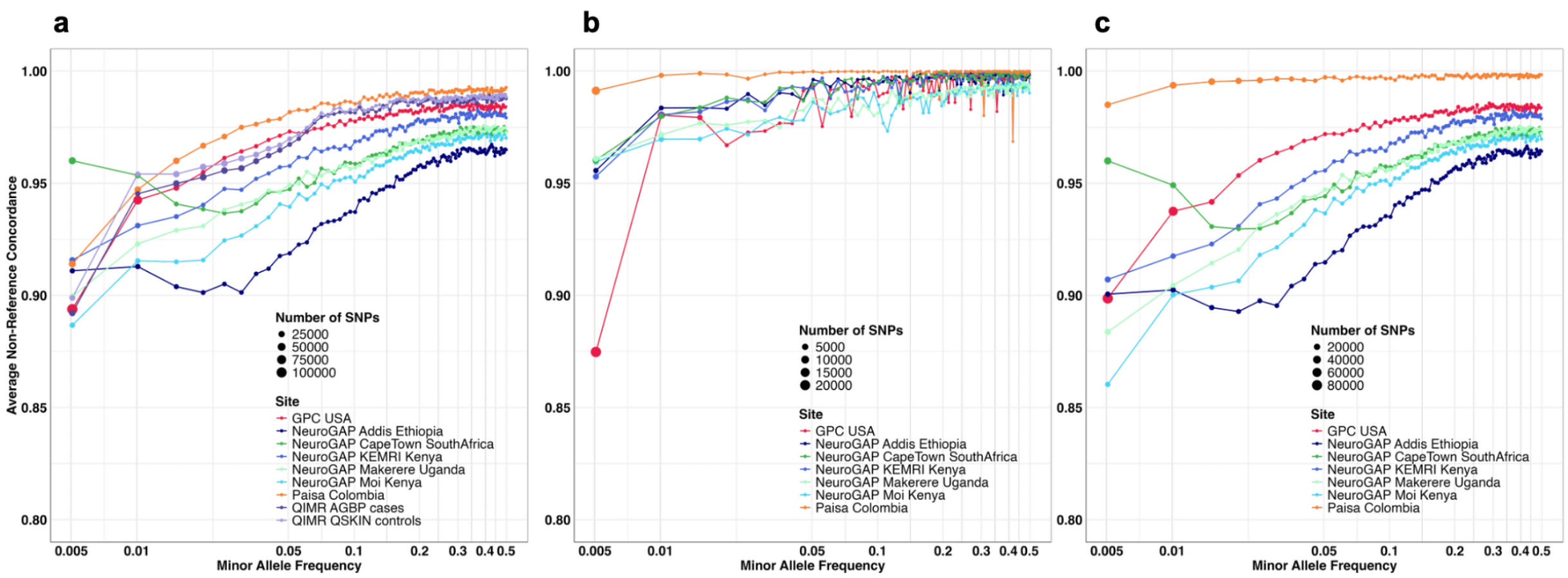

Supplementary Figure 6. Per-cohort non-reference concordance per minor allele frequency bin.

a. The x-axis provides the minor allele frequency bin while the y-axis provides the non-reference concordance per cohort. Size of the points corresponds to the number of SNPs in the MAF bin. Variants are filtered to those passing an INFO score >= 0.8. MAFs per SNP are defined by the GSA array data for the Paisa, GPC, and QIMR cohorts, given the sufficient sample sizes. MAFs are defined by the full cohort imputed data per site for the NeuroGAP sites.
b. Similar to above, average non-reference concordance for PUMAS cohorts, here using only exonic SNPs. Dips in the exonic SNP concordance are due to the very small number of SNPs falling within those bins (<100).
c. Average non-reference concordance for PUMAS cohorts, here using only non-exonic SNPs.

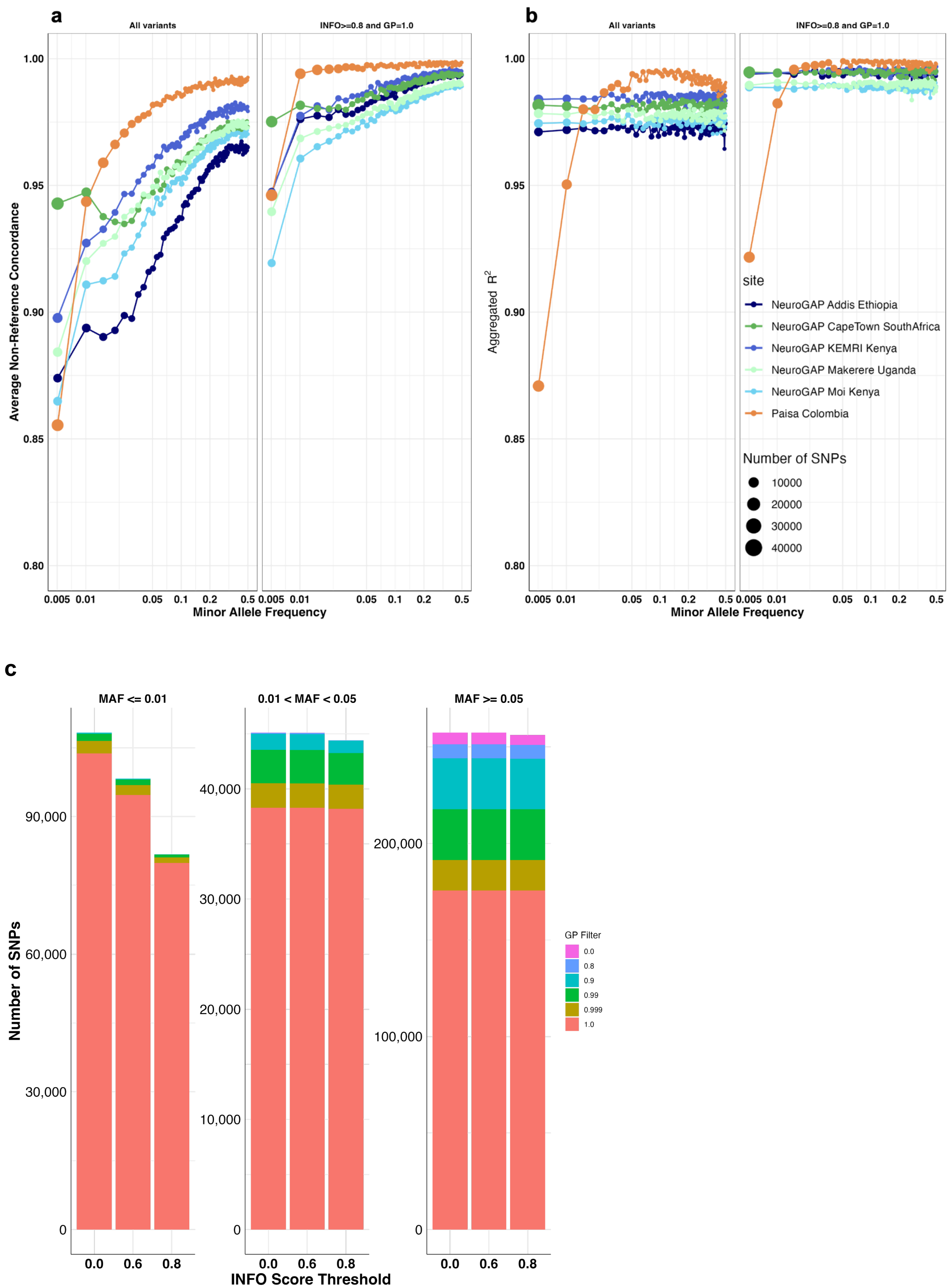
a
All variants
INFO>=0.8 and GP=1.0
Average Non-Reference Concordance
1.00
0.95
0.90
0.85
0.80
0.005 0.01 0.05 0.1 0.2 0.5
Minor Allele Frequency
b
Aggregated R^2
site
NeuroGAP Addis Ethiopia
NeuroGAP CapeTown SouthAfrica
NeuroGAP KEMRI Kenya
NeuroGAP Makerere Uganda
NeuroGAP Moi Kenya
Paisa Colombia
Number of SNPs
10000
20000
30000
40000
c
MAF <= 0.01
0.01 < MAF < 0.05
MAF >= 0.05
Number of SNPs
90,000
60,000
30,000
0
40,000
30,000
20,000
10,000
200,000
100,000
0.0 0.6 0.8
INFO Score Threshold
GP Filter
0.0
0.8
0.9
0.99
0.999
1.0

Supplementary Figure 7. Pre and post-QC imputation concordance and number of variants intersecting across the Illumina GSA array and imputed BGE data.

a. Non-reference concordance for the NeuroGAP and Paisa cohorts using unfiltered imputed data compared to filtering variants with INFO score at least 0.8 and requiring samples to have genotype probability (GP) = 1.0.
b. Aggregate R^2 for the NeuroGAP and Paisa cohorts using unfiltered imputed data compared to filtering variants with INFO score at least 0.8 and requiring samples to have genotype probability (GP) = 1.0.
c. Number of SNPs per INFO score filter and GP filter. SNPs were considered dropped by the GP filter if more than 90% of the samples did not reach the given threshold.

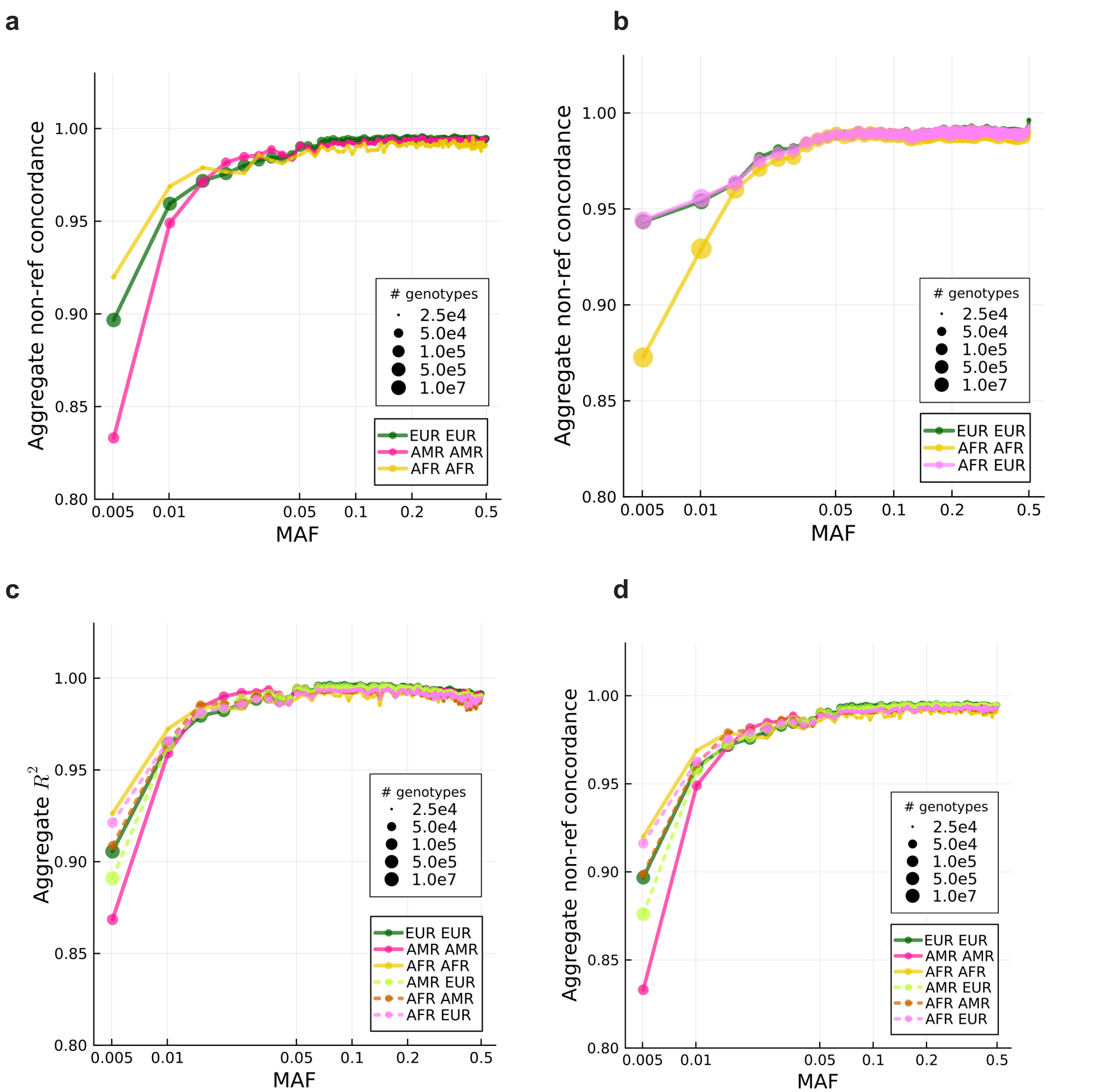

Supplementary Figure 8. Local ancestry imputation concordance.

a. Non-reference concordance for Paisa cohort.
b. Non-reference concordance for GPC cohort.
c. Aggregated R^2 for Paisa cohort, including heterozygous diploid ancestry (dashed lines).
d. Non-reference concordance for Paisa cohort, including heterozygous diploid ancestry (dashed lines).

Supplementary References

1. Fu, J.M., Satterstrom, F.K., Peng, M., Brand, H., Collins, R.L., Dong, S., Wamsley, B., Klei, L., Wang, L., Hao, S.P., et al. (2022). Rare coding variation provides insight into the genetic architecture and phenotypic context of autism. Nature Genetics *54*, 1320–1331.

2. McCarthy, S., Das, S., Kretzschmar, W., Delaneau, O., Wood, A.R., Teumer, A., Kang, H.M., Fuchsberger, C., Danecek, P., Sharp, K., et al. (2016). A reference panel of 64,976 haplotypes for genotype imputation. Nat Genet *48*, 1279–1283.

3. Lam, M., Awasthi, S., Watson, H.J., Goldstein, J., Panagiotaropoulou, G., Trubetskoy, V., Karlsson, R., Frei, O., Fan, C.-C., De Witte, W., et al. (2020). RICOPILI: Rapid Imputation for COnsortias PIpeLIne. Bioinformatics *36*, 930–933.

4. Rubinacci, S., Ribeiro, D.M., Hofmeister, R.J., and Delaneau, O. (2021). Efficient phasing and imputation of low-coverage sequencing data using large reference panels. Nat Genet *53*, 120–126.

5. Byrska-Bishop, M., Evani, U.S., Zhao, X., Basile, A.O., Abel, H.J., Regier, A.A., Corvelo, A., Clarke, W.E., Musunuri, R., Nagulapalli, K., et al. (2022). High-coverage whole-genome sequencing of the expanded 1000 Genomes Project cohort including 602 trios. Cell *185*, 3426–3440.e19.

6. Koenig, Z., Yohannes, M.T., Nkambule, L.L., Zhao, X., Goodrich, J.K., Kim, H.A., Wilson, M.W., Tiao, G., Hao, S.P., Sahakian, N., et al. (2024). A harmonized public resource of deeply sequenced diverse human genomes. Genome Res. *34*, 796–809.

7. Gurdasani, D., Carstensen, T., Tekola-Ayele, F., Pagani, L., Tachmazidou, I., Hatzikotoulas, K., Karthikeyan, S., Iles, L., Pollard, M.O., Choudhury, A., et al. (2015). The African Genome Variation Project shapes medical genetics in Africa. Nature *517*, 327–332.

8. Ramsay, M., Crowther, N., Tambo, E., Agongo, G., Baloyi, V., Dikotope, S., Gómez-Olivé, X., Jaff, N., Sorgho, H., Wagner, R., et al. (2016). H3Africa AWI-Gen Collaborative Centre: a resource to study the interplay between genomic and environmental risk factors for cardiometabolic diseases in four sub-Saharan African countries. Glob. Health Epidemiol. Genom. *1*, e20.
