# Supplementary material for: A blended genome and exome sequencing method captures genetic variation in an unbiased and cost-effective manner
Source: Nat Genet. 2026 Jul 8;58(8):1813–21. doi: 10.1038/s41588-026-02669-w (PMC13447102; doi:10.1038/s41588-026-02669-w)
Supplement: Supplementary file 2 [file 41588_2026_2669_MOESM2_ESM.pdf]

nature portfolio

Corresponding author(s): Alicia Martin; Daniel Howrigan, Benjamin Neale

Last updated by author(s): 03/19/2026 (by Toni Boltz)

Reporting Summary

Nature Portfolio wishes to improve the reproducibility of the work that we publish. This form provides structure for consistency and transparency in reporting. For further information on Nature Portfolio policies, see our Editorial Policies and the Editorial Policy Checklist.

Statistics

For all statistical analyses, confirm that the following items are present in the figure legend, table legend, main text, or Methods section.

n/a	Confirmed	
☐	☒	The exact sample size (*n*) for each experimental group/condition, given as a discrete number and unit of measurement
☐	☒	A statement on whether measurements were taken from distinct samples or whether the same sample was measured repeatedly
☐	☒	The statistical test(s) used AND whether they are one- or two-sided<br>*Only common tests should be described solely by name; describe more complex techniques in the Methods section.*
☒	☐	A description of all covariates tested
☒	☐	A description of any assumptions or corrections, such as tests of normality and adjustment for multiple comparisons
☒	☐	A full description of the statistical parameters including central tendency (e.g. means) or other basic estimates (e.g. regression coefficient) AND variation (e.g. standard deviation) or associated estimates of uncertainty (e.g. confidence intervals)
☒	☐	For null hypothesis testing, the test statistic (e.g. *F*, *t*, *r*) with confidence intervals, effect sizes, degrees of freedom and *P* value noted<br>*Give P values as exact values whenever suitable.*
☒	☐	For Bayesian analysis, information on the choice of priors and Markov chain Monte Carlo settings
☒	☐	For hierarchical and complex designs, identification of the appropriate level for tests and full reporting of outcomes
☐	☒	Estimates of effect sizes (e.g. Cohen's *d*, Pearson's *r*), indicating how they were calculated

Our web collection on statistics for biologists contains articles on many of the points above.

Software and code

Policy information about availability of computer code

Data collection

no software was used for data collection

Data analysis

https://github.com/atgu/bge_analysis

Software versions used:
Hail 0.2.128 for exome QC.
GATK 4.1.0.0 gCNV pipeline.
VISTA does not list specific versions (https://github.com/Mangul-Lab-USC/VISTA) we listed as v1 in this BGE manuscript
INSURVEYOR v1.1.3
Beagle 5.4 Phasing array genotypes for Local Ancestry Inference.
Beagle 5.1 Imputing Illumina GSA array data
GLIMPSE2 v2 Imputation of low-pass whole genome sequencing data.
RFMix2 v2 2-way and 3-way Local Ancestry Inference.

For manuscripts utilizing custom algorithms or software that are central to the research but not yet described in published literature, software must be made available to editors and reviewers. We strongly encourage code deposition in a community repository (e.g. GitHub). See the Nature Portfolio guidelines for submitting code & software for further information.

Data

Policy information about availability of data

All manuscripts must include a data availability statement. This statement should provide the following information, where applicable:

- Accession codes, unique identifiers, or web links for publicly available datasets
- A description of any restrictions on data availability
- For clinical datasets or third party data, please ensure that the statement adheres to our policy

We describe all datasets in the manuscript or its Supplementary Tables 1-2Information. Genome Reference Consortium Human Build 38 can be accessed at https://www.ncbi.nlm.nih.gov/assembly/GCF_000001405.40/. Data generated and analyzed come from several studies. In fulfillment of NIH data sharing policies, data used in this study has been made publicly available in NIMH Data Archive (NDA) Study Accessions #3805 (NDA-GRU), #4538 (Disease-Specific (Mental Health)), #4539 (NDA-HMB-MDS), dbGaP Study Accession phs001642.v1.p1, and phs002502.v3.p1. Sequence data available for each study accession include a CRAM file per individual as well as quality-controlled genomic variant data calls (vcf files) that are compatible with common software tools such as hail or bcftools.

For questions about accessing the QIMR dataset please contact Sarah Medland.

For NeuroGAP data, we follow H3Africa guidelines for data sharing which are designed to both recognize the challenges faced by researchers in Africa in making the best use of their data and the needs of the scientific community to share data. The principles are here and approved by the NIMH: https://www.sciencedirect.com/science/article/pii/S0168952514001966 In order to give African researchers time to publish on their data, the data will be shared publicly in the NDA in July 2027.

Research involving human participants, their data, or biological material

Policy information about studies with human participants or human data. See also policy information about sex, gender (identity/presentation), and sexual orientation and race, ethnicity and racism.

Reporting on sex and gender	Sex information was collected via self-report, and checked via imputation of genetic data.
Reporting on race, ethnicity, or other socially relevant groupings	Based on ancestry from genetic PCs as well as self-report.
Population characteristics	All cohorts include individuals diagnosed with severe mental illness, including bipolar disorder, schizophrenia, and depression as well as age/sex matched controls with no history of psychiatric illness.
Recruitment	All cohorts exclusively recruited participants who were 18 years of age or older at the time of enrollment.
Ethics oversight	Received approval from the Institutional Review Boards at each participating institution and recruitment site within PUMAS (Populations Underrepresented in Mental illness Association Studies), and all participants signed an informed consent form agreeing to be part of the study. This information is also included in the manuscript.<br><br>All cohorts involving human subjects that were included in this research have IRB approval following a full review of IRB applications and study consent forms by appropriate ethics committees. Details on each of the participating cohorts and IRB approvals are listed below:<br><br>Hispanic ASD samples, IRB title: The Study of Novel Autism Genes, Protocol #2012p001018, IRB of record: Mass General Brigham (previously known as PARTNERS), PI on IRB: Mark Daly<br><br>Chinese samples, IRB title: Neuropsychiatric genetics of a Chinese population from Shanghai Province, Protocol # IRB17-1379, IRB of record: Harvard T.H. Chan School of Public Health (HSPH), PI on IRB: Benjamin Neale<br><br>NeuroGAP-Psychosis:Ethiopia and South African samples, IRB title: Neuropsychiatric Genetics of African Populations - Psychosis (NeuroGAP-Psychosis), Protocol # IRB17- 0822, IRB of record: Harvard T.H. Chan School of Public Health (HSPH), PI on IRB: Karestan Koenen<br><br>Paisa and Genomic Psychiatry Cohort, IRB title: Molecular Profiling of Psychiatric Disease, Protocol #2014p001342, IRB of record: Mass General Brigham (previously known as Partners Healthcare), PI on IRB: Benjamin Neale<br><br>Inflammatory Bowel Disease samples, IRB title: The Broad Institute Study of Inflammatory Bowel Disease Genetics, Protocol # 2013P002634, IRB of record: Mass General Brigham (previously known as Partners Healthcare), PI on IRB: Ramnik Xavier<br><br>All participants who joined the studies listed under the above IRBs consented to the de-identified use of their data in disease research studies. During the consenting process, study staff explain the research goals and the basics of genetic processing activities. Participants are also given the option to opt out of studies if they choose. Samples used in this research and method development are all de-identified and have all been fully consented for inclusion in the study and any resulting publications.

Note that full information on the approval of the study protocol must also be provided in the manuscript.

Field-specific reporting

Please select the one below that is the best fit for your research. If you are not sure, read the appropriate sections before making your selection.

☐ Life sciences ☒ Behavioural & social sciences ☐ Ecological, evolutionary & environmental sciences

For a reference copy of the document with all sections, see nature.com/documents/nr-reporting-summary-flat.pdf

Behavioural & social sciences study design

All studies must disclose on these points even when the disclosure is negative.

Study description	The Populations Underrepresented in Mental illness Association Studies (PUMAS) project is attempting to remediate the historical underrepresentation of African and Latin American populations in psychiatric genetics through large-scale genetic association studies of individuals diagnosed with a serious mental illness [SMI, including schizophrenia (SCZ), schizoaffective disorder (SZA) bipolar disorder (BP), and severe major depressive disorder (MDD)] and matched controls.
Research sample	Individuals with history of psychotic illness (cases) or no psychiatric history (controls) from the following countries: United States (GPC) , Colombia (Paisa), South Africa, Kenya, Uganda, and Ethiopia (NeuroGAP)
Sampling strategy	The Genomic Psychiatry Cohort (GPC) is a multi-institutional collaboration led by Rutgers University. The GPC resource includes an NIMH-managed repository of genomic samples at SAMPLED, genotypic and sequence data, and detailed clinical and demographic data for investigations of schizophrenia, bipolar disorder, OCD, and COVID from a variety of ancestries collected in the USA. Within this analysis, we included 4,553 samples with 3,926 passing QC filters.<br><br>The Paisa population is a genetic isolate from Colombia that has expanded rapidly following a series of migration-related bottlenecks. They have been the focus of genetics studies in neuropsychiatric disorders in the last decade. Paisa BGE data generated here emerged from a long-standing collaboration between teams at the Universidad de Antioquia, Medellin, Colombia and University of California, Los Angeles to recruit samples from this population. Within the analysis, we included 9,007 samples with 8,200 passing QC filters.<br><br>The Stanley Center at the Broad Institute of MIT & Harvard initiated the NeuroGAP-Psychosis project in 2015, a collaboration with colleagues at Addis Ababa University in Ethiopia, KEMRI-Wellcome Trust in Kenya, Makerere University in Uganda, Moi University/ Moi Teaching and Referral Hospital in Kenya, the University of Cape Town in South Africa, and the Harvard T.H. Chan School of Public Health in the United States. With initial plans to recruit 35,000 participants (half cases with a diagnosis of schizophrenia or bipolar disorder and half controls), the target was expanded to 39,000 participants via the PUMAS Project awarded by NIMH. Sample recruitment ultimately exceeded the revised target, with >42,000 samples collected across the five NeuroGAP-Psychosis collection sites. Within the analysis, we included 39,886 samples with 35,138 passing QC filters.
Data collection	BGE data used in these analyses were generated at the Broad Clinical Lab. Sample cohorts included in the dataset were recruited and submitted from the collaborating institutions for the PUMAS project which includes cohorts from the GPC, Paisa population, and NeuroGAP-Psychosis.
Timing	Data collection began as early as October 2017 and ended in March 2023, with a pause from March 2020 – June 2020 due to COVID.
Data exclusions	For the GPC study, exclusion criteria included any premorbid organic mental disorders (i.e., epilepsy, CNS infection, significant head trauma, mental retardation), and premorbid history of significant drug or alcohol dependence by DSM IV/5 that confounds the diagnosis of schizophrenia.<br>Otherwise, data was excluded based on failing quality control filters for the sequencing data.
Non-participation	No participants dropped out.
Randomization	Participants were organized into cohorts based on country of origin, but no randomization within cohorts was necessary.

Reporting for specific materials, systems and methods

We require information from authors about some types of materials, experimental systems and methods used in many studies. Here, indicate whether each material, system or method listed is relevant to your study. If you are not sure if a list item applies to your research, read the appropriate section before selecting a response.

Materials & experimental systems

n/a	Involved in the study
☒	☐ Antibodies
☒	☐ Eukaryotic cell lines
☒	☐ Palaeontology and archaeology
☒	☐ Animals and other organisms
☒	☐ Clinical data
☒	☐ Dual use research of concern
☒	☐ Plants

Methods

n/a	Involved in the study
☒	☐ ChIP-seq
☒	☐ Flow cytometry
☒	☐ MRI-based neuroimaging

Plants

Seed stocks

Report on the source of all seed stocks or other plant material used. If applicable, state the seed stock centre and catalogue number. If plant specimens were collected from the field, describe the collection location, date and sampling procedures.

Novel plant genotypes

Describe the methods by which all novel plant genotypes were produced. This includes those generated by transgenic approaches, gene editing, chemical/radiation-based mutagenesis and hybridization. For transgenic lines, describe the transformation method, the number of independent lines analyzed and the generation upon which experiments were performed. For gene-edited lines, describe the editor used, the endogenous sequence targeted for editing, the targeting guide RNA sequence (if applicable) and how the editor was applied.

Authentication

Describe any authentication procedures for each seed stock used or novel genotype generated. Describe any experiments used to assess the effect of a mutation and, where applicable, how potential secondary effects (e.g. second site T-DNA insertions, mosiacism, off-target gene editing) were examined.
